# An Experimental and Theoretical Study of the Valence Shell Electronic Structure of Nitromethane

**DOI:** 10.1021/acs.jpca.5c04698

**Published:** 2025-09-17

**Authors:** Ivan Powis, Juliana Cuéllar-Zuquin, Angelo Giussani, Javier Segarra-Martí, Bérenger Gans, Ugo Jacovella, John D. Bozek, Stephen T. Pratt, David M. P. Holland

**Affiliations:** † School of Chemistry, 6123 The University of Nottingham, University Park, Nottingham NG7 2RD, U.K.; ‡ Instituto de Ciencia Molecular, 16781 Universitat de València, C/Catedrático José Beltrán 2, Paterna, Valencia 46980, Spain; § Institut des Sciences Moléculaires d’Orsay, CNRS, 173578 Université Paris-Saclay, Orsay F-91405, France; ∥ 55536 Synchrotron SOLEIL, l’Orme des Merisiers, Départmentale 128, Saint-Aubin 91190, France; ⊥ Chemical Sciences and Engineering Division, 1291 Argonne National Laboratory, Lemont, Illinois 60439, United States; # ASTeC, Science and Technology Facilities Council, Daresbury Laboratory, Warrington WA4 2DS, U.K.

## Abstract

Vibrationally resolved CH_3_NO_2_ and CD_3_NO_2_ photoelectron spectra and angular distribution parameters, β, have been measured in the photon energy range 20–80 eV. This allows a comprehensive investigation of conflicting literature interpretations of the two outermost photoelectron bands, complemented by high-level calculations characterizing the three O lone-pair-based orbital ionizations expected in this region. Franck–Condon simulations allow the regular vibrational progressions observed at the beginning of the first and second bands to be assigned as predominantly modes *v*_5_ and *v*_6_ of the D_0_ and D_2_ cation states, respectively. Irregular structuring observed midband in both bands is attributed to avoided crossings between the adiabatic D_0_/D_1_ and D_1_/D_2_ states as identified in potential surface cuts taken along the *v*_5_ and *v*_6_ normal mode coordinates. The associated D_0_/D_1_ and D_1_/D_2_ conical intersections were located and characterized, allowing possible vibronic coupling and its impact on the photoelectron spectra of the D_0_, D_1_, and D_2_ states to be discussed. This helps rationalize the apparently missing D_1_ ionization in this region, although some contribution by this state may be identifiable in the β-parameter spectra. Theoretical analysis of the complete valence region spectrum identifies the early breakdown of the independent electron model beyond these first two bands.

## Graphical abstract

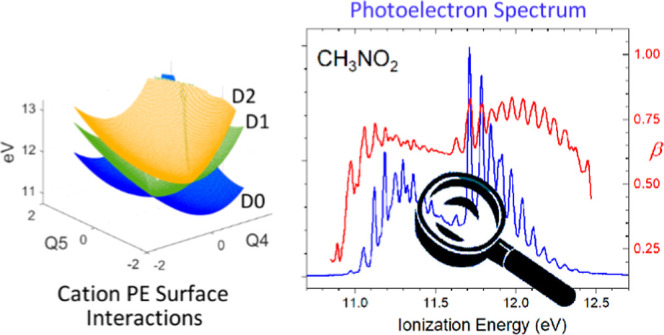

## 1 Introduction

The outer valence shell photoelectron spectrum of nitromethane (CH_3_NO_2_), first recorded by Rabalais^1^ using HeI radiation, exhibits six bands in the ionization range 11–20 eV. The two lowest energy bands, falling between 11 and 12.3 eV, displayed vibrational progressions with a spacing of ∼70 meV. The assignment of the outer valence photoelectron bands, and particularly the lowest two containing vibrational structure, remains uncertain, despite numerous experimental studies using NeI,^2^ HeI,^2–7^ HeII,^4,8^ and Al Kα^9^ radiation and Penning ionization.^5^ Perhaps surprisingly, all the reported photoelectron spectra have been measured using discrete line photon sources, rather than synchrotron radiation, and the original spectrum recorded in 1972 remains the best available.^1^

There have been several theoretical examinations of the outermost electronic structure of nitromethane.^2,4,5,8,10–14^ It is well established that the three outer molecular orbitals correspond to lone-pair combinations largely localized on the oxygen atoms, and all may be expected to have ionization energies that fall in the 11–12.3 eV range of the valence photoelectron spectrum. However, there is considerable variation in their assignment to the two experimental peaks observed in the relevant region. The ordering of the canonical molecular orbitals is now recognized to be very dependent on the method and basis set employed for calculations. Therefore, predicted ionization energies, which have often been simply inferred by implicit or explicit application of Koopmans theorem, are correspondingly at variance. Consequently, the assignment and interpretation of the first two structured photoelectron bands in terms of the three outer orbitals remain ambiguous and conflicting.

The presence of the “missing” third cation state in this energy region has, however, been inferred from two related experimental observations. Katsumata et al.^2^ measured the photoelectron angular distributions, as characterized by the anisotropy parameter β, using NeI and HeI radiation. A small, localized peaking of the β value, in the energy region between the two band maxima (at 11.3 and 11.7 eV), was attributed to an additional intermediate state that was not otherwise clearly identifiable in the photoelectron spectrum. On the other hand, Shastri et al.^13^ have examined the vacuum ultraviolet absorption spectrum of nitromethane and identified Rydberg series that appear to converge to an ionization limit at 11.95 eV, above the energy of the onset of the second photoelectron band. These two deduced energies for a possible third cationic state are thus not consistent, and so the overall assignment and interpretation of the low energy region of the photoelectron spectrum in terms of the electronic structure of nitromethane remains as an unresolved question.

The aim of the present experimental and theoretical study is to improve our understanding of the valence shell electronic structure of nitromethane. Various theoretical methods have been employed to calculate the outer valence orbital ionization energies, and the applicability of the single particle model of ionization^15^ has been investigated by generating the complete valence shell photoelectron spectrum using ionization energies and relative spectral intensities obtained from ADC(3) calculations.^16,17^ High-resolution, polarization-dependent, photoelectron spectra of CH_3_NO_2_ have been measured over a photon energy range 20–80 eV, thereby allowing the photoelectron angular distributions (β values) and the band intensities to be determined. These experimental results are compared to the corresponding theoretical predictions for branching ratios and β parameters obtained through the CMS-Xα approach.^18,19^

The vibrational structure in the two lowest energy photoelectron bands of CH_3_NO_2_ and deuterated CD_3_NO_2_ was also examined in detail. A comparison with Franck–Condon simulations employing harmonic vibrational modes has enabled some of the peaks near the beginning of the first and second photoelectron bands to be assigned to the excitation of specific vibrational modes. However, in each photoelectron band, a discontinuity occurs in the regular progressions, at which point the structure at higher energies becomes erratic. The onset of this irregularity is discussed in relation to possible vibronic interactions between the lowest cationic states, making use of calculated adiabatic potential cuts along the normal mode coordinates of nitromethane, and by characterizing the associated minimum energy conical intersections (MECI) and intersection seams connecting them.

## 2 Experimental Method

The photoelectron spectra were recorded with a VG Scienta R4000 hemispherical electron analyzer mounted on the soft X-ray undulator-based PLEIADES beamline at the SOLEIL synchrotron radiation facility. Detailed descriptions of the beamline and station instrumentation have been reported previously^20–22^ so only a summary is given here.

The undulator allows the electric vector of the linearly polarized synchrotron radiation to lie either parallel or perpendicular to the acceptance axis of the electron analyzer. The beamline employs a modified Petersen type monochromator. The spectra were recorded using gratings having 400 or 600 grooves/mm and exit slit widths that varied between 70 and 100 μm. The resulting theoretical optical resolution varied between ∼2 meV at a photon energy of 20 eV and ∼14 meV at 80 eV.

The electron spectrometer was mounted in a fixed position and photoionization occurred within an in-house-designed cell^23^ to minimize the throughput of the sample. The polarization dependent spectra were recorded with an analyzer pass energy of 10 eV and a 0.2 mm curved entrance slit, resulting in a theoretical spectrometer resolution of 5 meV. Some additional spectra encompassing only the two lowest energy photoelectron bands of CD_3_NO_2_ were recorded, mainly at low photon energies, using an analyzer pass energy of 5 eV and the same curved entrance slit, resulting in a theoretical spectrometer resolution of 2.5 meV. For these spectra, an optical resolution of 1–2 meV was used. Translational Doppler broadening contributes to the overall line width.^24^ For an electron kinetic energy of 28.7 eV, which corresponds to ionization into the lowest ionic state at hν = 40 eV, the Doppler width contribution is ∼12 meV.

The electron ionization energy scale was calibrated by recording a spectrum of a mixture of nitromethane and xenon, and using the Xe^+^^2^P_3/2_ and ^2^ *P*_1/2_ ionization energies of 12.130 and 13.436 eV, respectively.^25^

The liquid samples of CH_3_NO_2_ and CD_3_NO_2_ were subjected to several freeze–pump–thaw cycles prior to admitting some of the vapor in equilibrium with the room temperature sample into the chamber via a needle valve.

The electron angular distribution for photoionization of randomly oriented molecules using 100% polarized radiation is given by^26^

1 $$\frac{d\sigma }{d\Omega }=\frac{\sigma }{4\pi }\left[1+\beta P_{2}\left(\cos\theta \right)\right]$$

where σ is the angle-integrated cross section, θ is the angle between the momentum of the ejected electron and the electric vector of the linearly polarized radiation, β is a parameter characterizing the angular distribution, P_2_ is the Legendre polynomial of second degree, and dΩ is the differential solid angle element in the direction specified by the polar angle θ.

Equation 1 can be rearranged into the more convenient form

2 $$\beta =\frac{2\left(I_{par}-I_{perp}\right)}{I_{par}+2I_{perp}}$$

where *I*_par_ and *I*_perp_ are the normalized electron intensities for parallel and perpendicular polarization, respectively, relative to the electron detector axis. All the spectra were normalized to the sample pressure, the accumulation time, and the photon flux prior to processing. The spectra were corrected for the variation in the transmission efficiency of the electron analyzer as a function of the electron kinetic energy, as described by Jauhianinen et al.^27^

The experimental photoelectron anisotropy parameter was determined as described by Patanen et al.^28^ The analysis was performed by applying eq 2 point by point across the recorded spectra for each photon energy. After a mild smoothing was performed to reduce noise, the β spectrum clearly also reflects the vibrational structure evident in the photoelectron spectrum. This capability enables the dependence of the β-parameter on both the level of vibrational excitation and the corresponding electron kinetic energy to be examined.

## 3 Computational Procedures

### 3.1 Background Information

Nitromethane is commonly discussed as having two stable conformers,^13,29,30^ corresponding to the staggered or eclipsed configurations of the NO_2_ and CH_3_ moieties. However, the torsional barrier is very small, so the NO_2_ and CH_3_ groups can be considered to freely rotate around the C–N bond in a gaseous room-temperature sample. We henceforth ignore any coupling of the torsional motion,^29–31^ in effect allowing it to be considered as instantaneously frozen at any arbitrary angle during a calculation.

All calculations for ionization energies and harmonic vibrational frequencies reported here were performed for both staggered and eclipsed configurations (C_*s*_ symmetry) and many were repeated for other intermediate configurations (C_1_ symmetry) with torsion angles stepped by 5°–10°. In all cases, the neutral ground-state geometries were fully optimized as appropriate, apart from fixing a selected torsion angle.

### 3.2 Ionization Energies

Vertical ionization energies were calculated using the Outer Valence Green’s Function method (OVGF)^17,32^ implemented in Gaussian 16,^33^ the non-Dyson third-order algebraic-diagrammatic construction scheme (IP-ADC(3)),^16,17,34–37^ and coupled cluster methods (EOM-IP-CCSD,^38–41^ EOM-IP-CC­(2,3)^42,43^ ) implemented in Q-Chem 5.4.^44^ All calculations were made using the cc-pVTZ basis set and its corresponding augmented basis set with added diffuse functions, and employed a MP2/cc-pVTZ optimized neutral geometry. Additionally, the EOM-IP-CCSD and EOM-IP-CC­(2,3) calculations were repeated with the augmented and nonaugmented cc-pV*x*Z (*x* = D,Q) basis sets from Dunning’s correlation-consistent family,^45^ permitting extrapolation to the complete basis set (CBS) limit for improved energy estimates.^46^

The IP-ADC(3) calculations were used in two differing contexts. When seeking accurate vertical ionization energies for just a few outer valence ionizing transitions we chose the improved IP-ADC­(3­[4+]) variant^44^ using a third-order ground state density and the correspondingly improved self-consistent Σ­(4+) method.^47,48^ The Σ­(4+) procedure iteratively calculates the self-energy (Σ) up to fourth-order perturbation theory and beyond, ensuring that the electron correlation effects are consistently accounted for throughout the calculation.

The second purpose was to obtain a wide-ranging survey of the full valence region photoelectron spectrum. This necessitates computing many hundred transitions and was made more manageable by exploiting the symmetry of the staggered and eclipsed conformations to block the matrices and by using the resolution-of-identity (RI) approximation. Additionally, it proved beneficial to relax the convergence tolerance for the Davidson diagonalization procedure to 10^–5^, albeit with some loss of accuracy. In this manner, we were able to obtain IP-ADC(3) transitions spanning ionization energies to 39 eV.

### 3.3 Photoionization Cross Sections and Photoelectron Angular Distributions

Photoionization cross sections and electron anisotropy parameters, β, were calculated using an independent electron, continuum multiple scattering model with an Xα exchange potential (CMS-Xα).^18^ Our implementation has been described previously^49,50^ and is briefly summarized here with pertinent details.

The method involves constructing a self-consistent neutral molecule potential modeled as overlapping spherical regions centered on each atomic site, enclosed within a spherically symmetric outer sphere that extends to infinity. A self-consistent effective one-electron potential is generated, representing the exchange contributions using Slater’s Xα local density approximation.^51^ One-electron continuum functions are found from the potential model, after adaptation to ensure the correct asymptotic ion-electron Coulombic behavior, by solving the scattering problem using a symmetry-adapted basis of spherical harmonic angular functions. Hence, electric dipole photoionization matrix elements can be constructed. Finally, cross sections and β-parameters are calculated from the matrix elements using standard formulations.^18^

For these calculations on nitromethane (staggered and eclipsed) overlapping Norman atomic sphere radii were used,^52^ scaled by a factor of 0.84, and nuclear coordinates were again taken from a MP2/cc-pVTZ geometry optimization. An angular basis of spherical harmonics with *l*_max_ = 6; 3; 1 was used in, respectively, the outer sphere; the C and N atomic regions; and the H atom regions. For evaluation of the continuum function, these limits were increased to *l*_max_ = 12; 6; 4.

### 3.4 Franck–Condon Vibrational Simulations

Franck–Condon vibrational simulations for the photoelectron spectrum of the first three cation states were performed using the adiabatic Hessian model with the Duschinsky rotation in the ezFCF program.^53^ The necessary normal mode vibrational analyses were performed using QChem 5.4 ^44^ at optimized B3LYP/cc-pVTZ staggered and eclipsed geometries. These optimizations proceeded straightforwardly for the neutral and ground state cations (respectively restricted and unrestricted open shell calculations). Searches for fully optimized stationary geometries of the excited cation states using TDDFT at the same level were, however, problematic. A key issue appeared to be the switching of the electronic state between the optimization cycles. As an alternative approach, the excited states were calculated and optimized by the Maximum Overlap Method (MOM).^54^ In the context of a cation calculation this works by selecting the orbital from which an electron is to be ionized from among the neutral’s canonical orbitals at the start of the calculation. This “hole” configuration is maintained at each SCF cycle, avoiding variational collapse of the wave function back to the ground state cation. This MOM model is here fully consistent with the strong single-electron character of the low energy ionizations identified by the more sophisticated calculations discussed later in this work. Results using the B3LYP functional were fully corroborated by repeating these optimizations with the alternative PBE0 functional.

Once the state geometry is optimized, the Hessian is calculated and the vibrational analysis for the normal mode coordinates and frequencies then completed at the same B3LYP/cc-pVTZ level with an ultrafine SG-3 integration grid, for both deuterated CD_3_NO_2_ and undeuterated CH_3_NO_2_. Before further use, the calculated harmonic frequencies were scaled by a factor of 0.97 as recommended for this model chemistry.^55^ The lowest frequency mode in all cases was reported as ∼30 cm^–1^ but was often also imaginary, indicating an unbound motion. On examination, this mode is clearly the torsional mode in nitromethane. As noted at the start of this section, the internal rotation barrier is anticipated to be no more than a few cm^–1^ and, in relation to the computational accuracy available here, the torsional potential is effectively flat, hence the small imaginary “frequencies”. In any case, such a torsion would be far from the harmonic oscillator model, and direct observation of a vibration/rotation with such small quanta would not be expected at the resolution employed in the present experiment. Therefore, the torsional mode has been omitted from the ezFCF vibrational simulations.

The ezFCF simulations assumed a temperature of 300 K, matching the experimental sample temperature, to try to capture any hot-band structure. The output, in the form of a stick spectrum of transition energies and intensities, could finally be folded with a Gaussian broadening function, FWHM of 12 meV, to achieve a realistic simulation.

### 3.5 Multiconfigurational Potential Surface Calculations

Multiconfigurational electronic structure theory computations were carried out with the OpenMOLCAS package.^56^ The complete active space self-consistent field (CASSCF)^57^ method was used throughout. The active space (see Figure S1, Supporting Information) included all valence π/π* and occupied *n*_O_ lone-pair orbitals, as well as all 3σ and 3σ* molecular orbitals localized on the C–N and N–O bonds, leading to 14 electrons in 11 orbitals^58^ for the neutral ground state and 13 electrons in 11 orbitals for the cation electronic states. An equal-roots state average of 4 states for the neutral and 3 states for the cation calculations was used throughout. We used an Atomic Natural Orbital basis set with a large contraction (ANO-L)^59,60^ in its double-ζ polarized contraction (VDZP) coupled with the atomic compact Cholesky decomposition^61^ technique to speed up the computation of two-electron integrals.^62^ Subsequently, the complete active space second-order perturbation theory (CASPT2)^63,64^ in its rotated (RMS)^65,66^ multistate extension was applied on top of the previously defined CASSCF wave functions to obtain the desired energies.^67^

The ground and electronically excited state cation minima were optimized using RMS-CASPT2, as recently implemented in OpenMolcas.^56^ Two types of calculations were performed to characterize the conical intersections: (i) unconstrained MECI optimizations were obtained for both the staggered and eclipsed conformations, and for both the *n*_O_^–^/*n*_O_^+^ and *n*_O_^+^/*n*_O_^π^ degeneracies; and (ii) constrained optimizations were performed to characterize the C–N intersection seam for both the *n*_O_^–^/*n*_O_^+^ and *n*_O_^+^/*n*_O_^π^ degeneracies, starting from the more stable staggered configuration.

To compute the potential energy surfaces along each vibrational normal mode, a Python script was used to read the displacement vector of each mode in the neutral ground state. Six different scaling factors (0.5, 1.0, and 1.5 denoted by *q*) in both positive (i.e., adding to the reference geometry) and negative (i.e., subtracting for the reference geometry) directions were applied to obtain six geometries along each normal mode coordinate, with the value 0 corresponding to the global (staggered) ground state minimum geometry. A single-point RMS-CASPT2 calculation was performed on top of each of these geometries, resulting in a series of data points along each vibrational coordinate. These data points were then fitted to a fourth-order polynomial for plotting.

## 4 Results and Discussion

### 4.1 Structure of Neutral CH_3_NO_2_

A peculiarity of nitromethane is the extremely low barrier to internal rotation of the CH_3_ and NO_2_ groups around the C–N bond. Experimentally, the barrier height has been inferred as ∼2 cm^–1^.^68^ Theoretical calculations, for example, at the HF/6–31++G­(p,d) level,^29^ provide similar results, while more recent DFT calculations (PBE0/aug-cc-pVQZ)^13^ predict a 4 cm^–1^ barrier height. MP2/aug-cc-pVQZ calculations performed in the course of the present work indicate barrier heights of ∼1 cm^–1^ (rising to 16 cm^–1^ in the ${\mathrm{X̃}}^{+}$ cation). In all these calculations, a staggered conformation has the lower energy, but with such low barriers, unhindered internal rotation can be assumed in a room-temperature gas phase sample, passing freely through the extremities of staggered and eclipsed conformations. Both of these extreme conformers belong to the C_*s*_ point group. In the staggered conformer, the symmetry plane lies perpendicular to the NO_2_ group and bisects the ONO angle, while in the eclipsed conformer the symmetry plane contains the NO_2_ group. At all intermediate torsion angles, the molecule lacks any symmetry elements (C_1_ point group).

Nitromethane has 16 two-electron orbitals, and Figure 1 shows the energetically ordered Hartree–Fock cc-pVTZ canonical molecular orbitals 9–16 (where 16 is the HOMO) in the staggered and eclipsed forms and at an intermediate torsion angle. For the C_*s*_ symmetry staggered and eclipsed forms, the orbitals can be classified (and numbered) as a′ and a″ as marked in the figure, but because of the changed orientation of the symmetry plane, there is some partial switching a′ ↔ a″ between clearly corresponding orbitals in the staggered and eclipsed geometry. Referring to the orbitals by just their simple sequence number, as for the intermediate conformer, avoids potential confusion.

**Figure 1:**
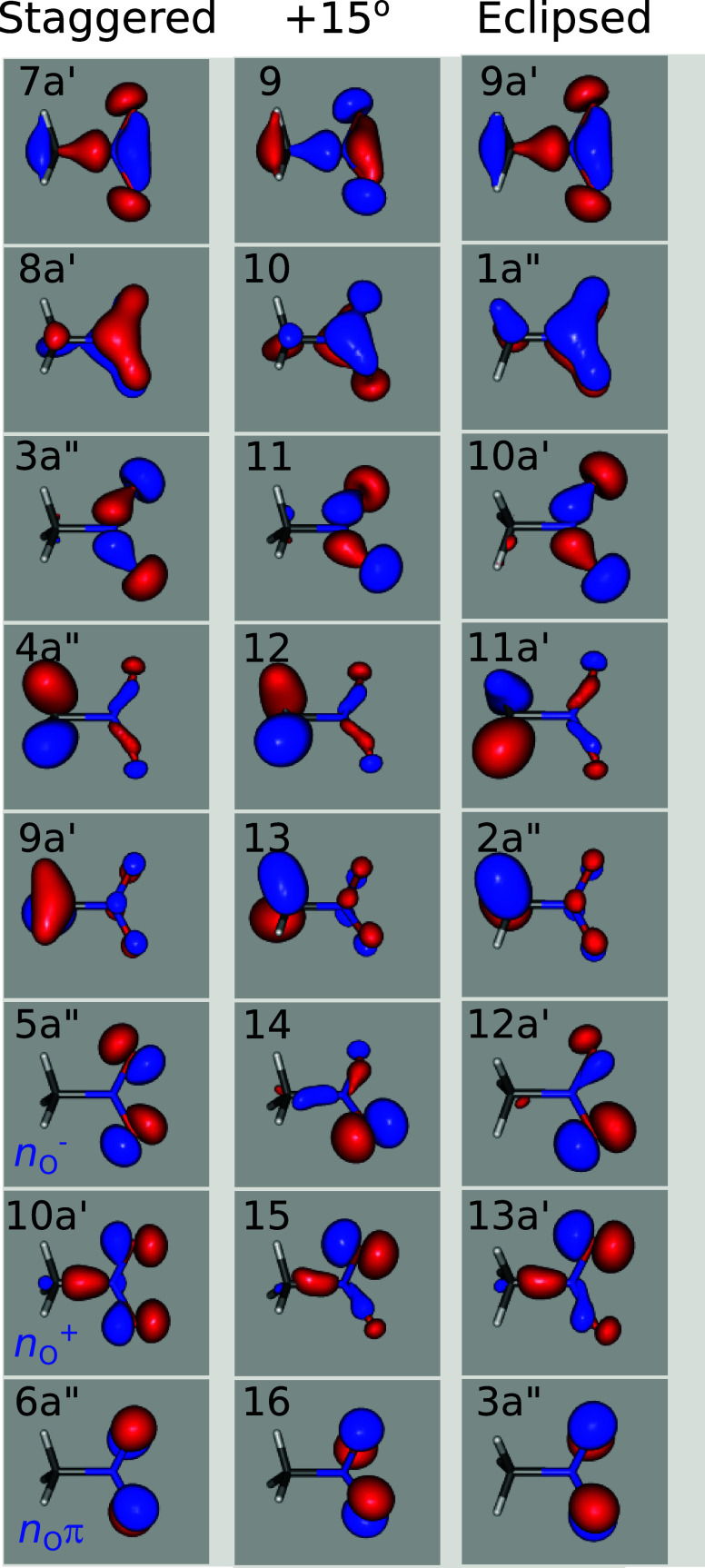
HF/cc-pVTZ canonical molecular orbitals for different torsional conformers of nitromethane. Symmetry classifications are indicated for the C_*s*_ symmetry staggered and eclipsed forms.

Table 1 lists the energies for the orbitals plotted in Figure 1 (plus the remaining orbitals 1–8) and it can be seen that the energies do not depend significantly on the conformation. From Figure 1 it may be seen that the characteristics of orbitals 9–13 and 16 are clearly preserved at all torsion angles, as are their energies (Table 1) but this equivalence is less immediately apparent for orbitals 14 and 15. These outer three orbitals (14–16) are predominantly comprised of lone pair orbitals localized on the NO_2_ grouping, with orbitals 14 and 15 consisting of combinations of the in-plane oxygen *p* orbitals. These are most naturally viewed in the staggered conformation where the orbitals 10a′ (15) and 5a″ (14) have been labeled, respectively, as the in-phase *n*_O_^+^ (with some additional C–N σ bonding character) and the antiphase combination *n*_O_^–^ (Mulliken populations are *n*_O_^+^ 10a′: O_1,2_-*p* = 0.3832, C-*p* = 0.1092 and *n*_O_^–^ 5a″: O_1,2_-*p* = 0.4599). From Table 1 these two orbitals are seen to be quasi-degenerate but of different symmetry in the staggered conformer. As the NO_2_ plane rotates with the changing torsion angle, the molecular symmetry distinction is lost and the in–NO_2_–plane O*p* atomic orbitals re-mix, slightly lifting the degeneracy between orbitals 14 and 15.

**Table 1:** HF/cc-pVTZ Canonical Orbital Energies (Hartrees) for Staggered, Intermediate, and Eclipsed Conformations

staggered		intermediate (staggered +15°/eclipsed −15°)		eclipsed	
orbital	energy	orbital	energy	orbital	energy
1a″	–20.605	1	–20.606	1a′	–20.607
1a′	–20.605	2	–20.604	2a′	–20.603
2a′	–15.852	3	–15.852	3a′	–15.852
3a′	–11.31	4	–11.31	4a′	–11.31
4a′	–1.602	5	–1.602	5a′	–1.602
2a″	–1.399	6	–1.399	6a′	–1.399
5a′	–1.105	7	–1.104	7a′	–1.104
6a′	–0.87	8	–0.87	8a′	–0.87
7a′	–0.753	9	–0.753	9a′	–0.753
8a′	–0.735	10	–0.735	1a″	–0.735
3a″	–0.734	11	–0.734	10a′	–0.734
4a″	–0.627	12	–0.627	11a′	–0.627
9a′	–0.601	13	–0.601	2a″	–0.601
5a″	–0.495	14	–0.496	12a′	–0.496
10a′	–0.495	15	–0.494	13a′	–0.494
6a″	–0.455	16 (HOMO)	–0.455	3a″	–0.455

We note that the energetic ordering of the outer canonical orbitals can be different with smaller basis sets or in a DFT calculation (see, e.g., ref 29), so that when comparing calculations in the literature, identification of the orbitals by their energetic sequence number has to be used with some caution. For discussing states derived from ionization of the outer three orbitals, the local NO_2_ C_2v_ symmetry descriptors *n*_O_^π^, *n*_O_^+^ (/C–N σ), *n*_O_^–^ are especially unambiguous in the staggered conformer, independent of their relative energetic ordering.

### 4.2 Overview of the Complete Valence Shell Photoelectron Spectrum

The complete valence shell photoelectron spectrum of nitromethane, recorded at a photon energy of 80 eV, is plotted in Figures 2 and S2 (Supporting Information). Sharp resolved bands in the threshold region around 11 eV are replaced by increasingly broad, undulating features in the inner valence region that eventually reduce to a low intensity continuum at ionization energies above 45 eV. This continuum is a consequence of the breakdown of the single particle model of ionization,^15^ enabling 1*h* configurations to interact with 2*h*1*p* configurations through electron correlation. As a result of this interaction, some of the intensity associated with the 1*h* states may be redistributed among numerous satellite states.

**Figure 2:**
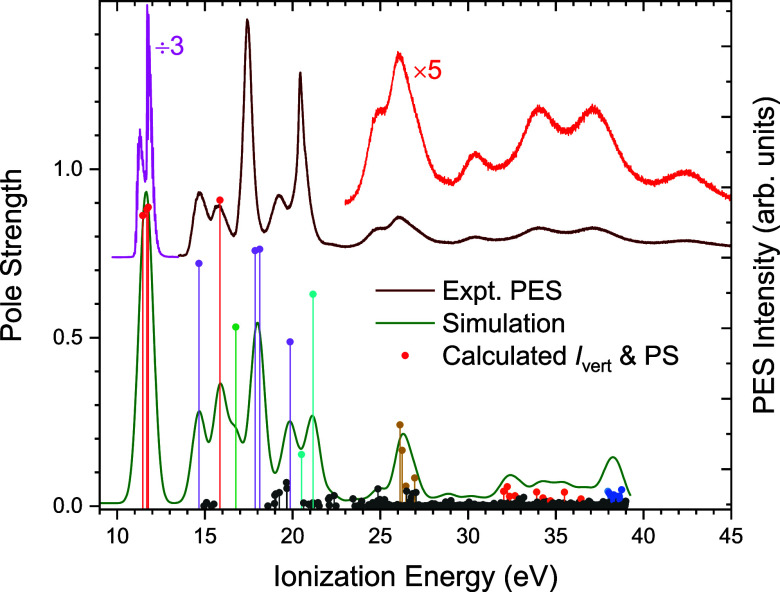
Photoelectron spectrum of nitromethane recorded at a photon energy of 80 eV, with the electron vector of the linearly polarized synchrotron radiation lying parallel to the electron detection axis. IP-ADC(3)/cc-pVTZ results for the staggered conformation are shown as a stick spectrum (ionization energies vs pole strength (PS)) and as a simulated spectrum profile that is obtained by convolution with a FWHM 900 meV Gaussian broadening function. Individual ionizations are color coded according to the nature of the excited state: red – 1*h*; purple – mixed 1*h*/2*h*1*p*; green – mixed 1*h*; cyan – 1*h*(8^–1^)/2*h*1*p*; yellow – 1*h*(7^–1^)/2*h*1*p*; orange -1*h*(6^–1^)/2*h*1*p*; blue -1*h*(5^–1^)/2*h*1*p*; black – all other mixed 2*h*1*p* states.

The IP-ADC(3) method^16,17,34–37^ extends beyond the independent electron one-hole (1*h*) model to include two-hole-one-particle (2*h*1*p*) transitions. By taking into account configuration interaction between the various 1*h* and 2*h*1*p* transitions in the wave function, ionic states with a mixed character may be qualitatively reproduced. These 2*h*1*p* contributions are known to be essential for the interpretation of the more complex inner valence region, where many satellite transitions are anticipated. The pole strengths derived in these calculations reflect the 1*h* character of the final states and offer a useful further diagnostic to aid interpretation.

Figure 2 shows calculated energies/pole strengths and a simulated spectrum obtained by convoluting the pole strengths with a Gaussian broadening function of 900 meV (FWHM). This matches the experimentally observed widths for the features at higher binding energies, facilitating comparison with the experimental spectrum. While the theoretical results plotted were obtained for the staggered conformation, the simulations obtained for the other conformers are effectively indistinguishable. Overall, the theoretical spectra display reasonable agreement with the experimental spectrum, thereby allowing an interpretation of the observed features.

The calculated ionizations up to 21 eV are listed in Table 2. The single particle model of ionization^15^ expected for the outer valence region clearly holds for the first three ionizations (16^–1^, 14^–1^, and 15^–1^) as indicated by pole strengths >0.86 in Table 2 (although note the reversed order of the 14^–1^ and 15^–1^ ionizations). However, even by the fourth ionization, dominantly 13^–1^, a mixed 1*h*/2*h*1*p* character shows the independent electron model weakening. Continuing to higher energies, the 12^–1^ ionization at 15.86 eV is again a strong single particle ionization. The adjacent 16.76 eV ionization has a mixed 10^–1^/13^–1^ 1*h* characteristic, but it is not readily identifiable in the experimental spectrum. Around 18 eV, the 11th and 12th transitions with strong pole strengths of ∼0.76 are dominantly single electron 11^–1^ and 9^–1^ ionizations; nevertheless, both have a small 2*h*1*p* character mixed in. Similarly, transition 20 clearly corresponds to a 10^–1^ single electron ionization with, nevertheless, an increased admixture of a 2*h*1*p* character.

**Table 2:** IP-ADC­(3)/cc-pVTZ Ionizing Transitions below 21 eV Listing Ionization Energy, Pole Strength (PS), and the Two Most Prominent configurations in the Respective Wavefunctions with |Amplitudes| ≥ 0.1^a^

			configuration I				configuration II			
transition	*I*_vert_ (eV)	PS	ampl	*i*	*j*	*v*	ampl	*i*	*j*	*v*
1	11.46	0.863	–0.95	16β						
2	11.70	0.880	0.95	14β						
3	11.78	0.887	–0.95	15β						
4	14.66	0.720	0.84	13β			0.30	16α	16β	17α
5	14.95	0.000	–0.48	15α	15β	17α	0.43	14α	14β	17α
6	15.10	0.010	0.48	15α	16β	17α	0.25	16α	15β	17α
7	15.37	0.000	0.54	15β	14β	17β	–0.27	14α	15β	17α
8	15.51	0.006	0.46	14α	16β	17α	0.23	16α	14β	17α
9	15.86	0.908	0.96	12β						
10	16.76	0.531	0.57	10β			0.46	13β		
11	17.86	0.758	–0.87	11β			–0.17	15β	16β	17β
12	18.13	0.762	–0.87	9β			–0.16	16α	14β	17α
13	18.60	0.000	0.36	15α	14β	17α	0.36	14α	15β	17α
14	18.97	0.008	–0.51	13α	16β	17α	–0.29	13β	16β	17β
15	18.99	0.032	–0.49	12α	16β	17α	–0.25	16α	12β	17α
16	19.05	0.036	–0.32	14α	14β	17α	–0.30	15α	15β	17α
17	19.24	0.040	–0.36	13α	14β	17α	–0.31	15β	16β	17β
18	19.65	0.070	–0.30	14α	13β	17α	0.29	13β	14β	17β
19	19.68	0.053	–0.33	13α	15β	17α	–0.33	14β	16β	17β
20	19.86	0.488	–0.69	10β			–0.33	16α	16β	17α
21	20.51	0.153	0.34	8β			0.31	11α	16β	17α
22	20.63	0.009	0.44	12α	14β	17α	0.44	12β	14β	17β
23	20.92	0.005	0.49	14α	12β	17α	0.26	12α	14β	17α

a For each configuration *i* (*j*) indicates an excited orbital for a one (two)-hole excitation and, in the case of 2*h*1*p* terms, *v* is an excited virtual orbital; α and β distinguish up and down electron spin states.

All of the transitions explicitly mentioned in the preceding paragraph have a significant single electron 1*h* component and are consequently identifiable from their large pole strengths >0.5. Color coding has been applied in Figure 2 to help distinguish those 1*h* transitions that nevertheless have a mixed character with either a second 1*h* configuration (plotted in green) or a 2*h*1*p* term (in purple)—either being indicative of some electron correlation. It is also apparent in Figure 2 and Table 2 that there is a small cluster of predicted mixed 2*h*1*p* transitions around 15 eV due to ionization accompanied by an excitation to the LUMO, orbital 17. However, these transitions are vanishingly weak and are not identifiable in the experimental spectrum. A further cluster of mixed 2*h*1*p* transitions that appears around 19–20 eV is predicted with a finite strength that could allow their experimental observation, although the present spectra are too congested to confirm this prediction. Nevertheless, these calculated results strongly suggest that the single molecular orbital picture of ionization^15^ is already compromised below 20 eV.

In Table 3 we have extracted various shakeup satellite transitions. Those falling around 19–20 eV contain a strong main-line component (11^–1^, 9^–1^, 10^–1^) and were already noted above. At slightly higher energies sits a pair of nominally 8^–1^ transitions (21 and 26), the latter at 21.17 eV retaining a strong one electron character (pole strength 0.63). This may then be considered the main-line, and the much weaker (pole strength of 0.153) ionization at 20.51 eV is formally a “shake-down” satellite. Common to all these, and indeed to all the other 2*h*1*p* components listed in Table 2, one may note that the shakeup state is accessed by populating orbital 17 (LUMO). This is an NO_2_ π* orbital, shown in Figure 3a.

**Table 3:** IP-ADC­(3)/cc-pVTZ Predicted Transitions with a Strong Mainline and Coupled 2*h*1*p* Shakeup^a^

			main line		shake-up configuration			
transition	*I*_vert_ (eV)	PS	ampl	*i*	ampl	*i*	*j*	*v*
11	17.86	0.758	–0.87	11β	–0.17	15β	16β	17β
12	18.13	0.762	–0.87	9β	–0.16	16α	14β	17α
20	19.86	0.488	–0.69	10β	–0.33	16α	16β	17α
21	20.51	0.153	0.34	8β	0.31	11α	16β	17α
26	21.17	0.629	–0.80	8β	0.22	15α	13β	17α
68	26.13	0.241	0.49	7β	–0.21	15α	15β	21α
70	26.25	0.165	–0.41	7β	0.25	14α	12β	18α
73	26.46	0.059	0.24	7β	–0.23	15α	13β	18α
83	26.96	0.084	0.29	7β	0.23	15α	12β	19α
214	32.03	0.044	–0.21	6β	0.22	10β	16β	21β
222	32.24	0.057	–0.24	6β	0.15	7α	16β	17α
227	32.37	0.029	0.17	6β	0.15	12β	16β	23β
241	32.68	0.031	–0.17	6β	0.13	13β	16β	26β
299	33.91	0.042	0.20	6β	–0.12	12α	10β	21α
317	34.27	0.024	–0.15	6β	0.13	13α	15β	25α
375	35.50	0.041	–0.20	6β	–0.12	14α	13β	31α
425	36.44	0.021	0.15	6β	0.12	15α	15β	32α
520	37.97	0.044	0.21	5β	0.14	9β	12β	19β
523	38.03	0.039	0.20	5β	0.12	9β	15β	22β
530	38.10	0.034	–0.18	5β	–0.11	14α	13β	29α
545	38.30	0.016	–0.12	5β	–0.11	11α	11β	24α
546	38.31	0.033	0.18	5β	0.12	9β	14β	23β
552	38.42	0.025	0.16	5β	0.12	9β	14β	23β
561	38.55	0.029	0.17	5β	–0.11	13β	12β	25β
568	38.62	0.029	0.17	5β	0.09	10β	16β	23β
569	38.65	0.020	0.14	5β	0.14	13β	14β	29β
576	38.76	0.049	–0.22	5β	–0.10	10β	13β	22β

a Spin-up and spin-down electrons are distinguished using α and β.

**Figure 3:**
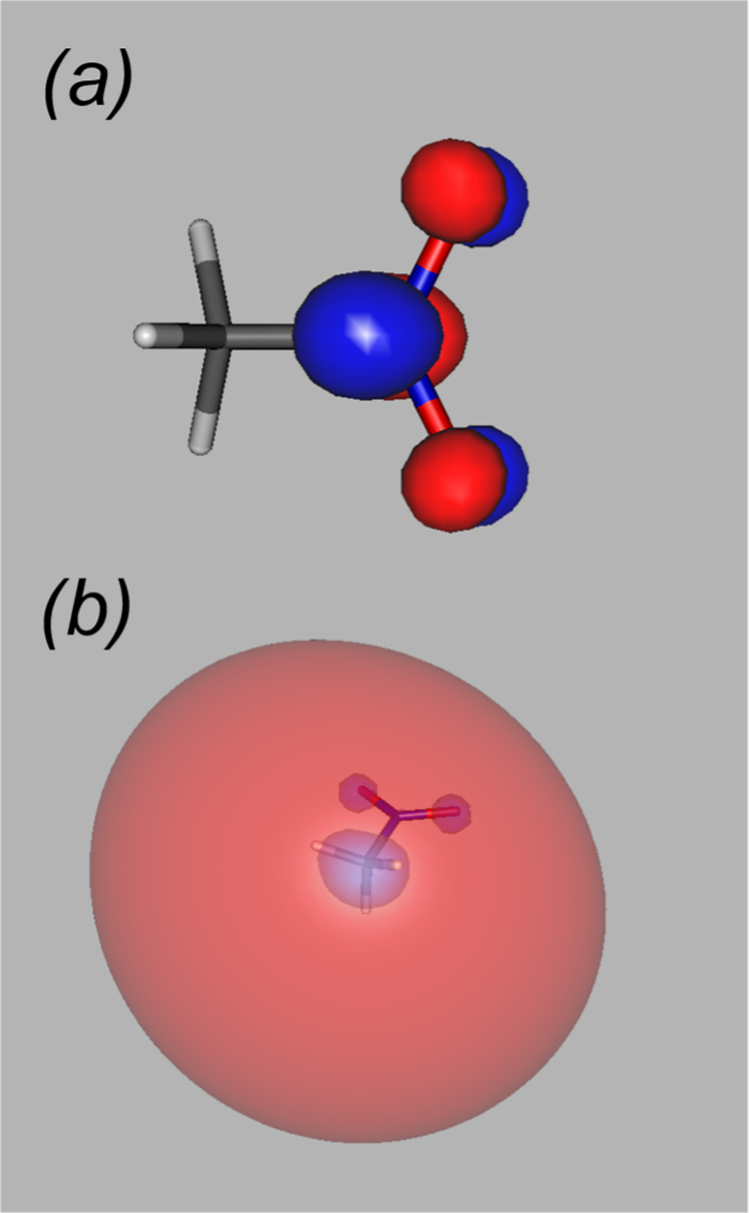
Virtual orbitals of staggered nitromethane computed with the aug-cc-pVTZ basis: (a) the LUMO [NO_2_ π*]; (b) the 3s Rydberg orbital.

In the inner valence region, one further anticipates that stronger electron correlation will result in the intensity associated with an inner valence orbital being redistributed among numerous satellites of low intensity and an absence of main-lines.

A grouping of 7^–1^ satellite transitions falls around 26–27 eV and is clearly responsible for a corresponding peak in the experimental spectrum at this position (Figure 2). Around 32–37 eV a similar clustering of 6^–1^ satellites can be seen in the ADC(3) simulation and may be associated with the experimental feature at 30.5 eV. To higher energy still, around 38 eV (the upper limit reached by these calculations), a cluster of 5^–1^ satellites appears. These 7^–1^, 6^–1^, and 5^–1^ satellites now involve excitation into a range of virtual orbitals above the π* LUMO. For example, orbital 21 accessed in 5^–1^ and 6^–1^ satellite clusters is a Rydberg 3s. This diffuse character is not reliably reproduced without added diffuse functions in the basis set, and the full calculation was therefore repeated using the aug-cc-pVTZ basis. The 3s orbital, for example, now falls below the energy of the initial π* LUMO. However, there is also a systematic small increase in all the orbital energies (discussed in the following section) and so, apart from an overall 0.2 eV energy shift, the aug-cc-pVTZ simulated profile of the photoelectron spectrum using a 900 meV Gaussian smoothing function is virtually indistinguishable from that of the un-augmented calculation.

Tables 2 and 3 list only the very small number of 1*h*, mixed 1*h*, and 1*h*/2*h*1*p* shake-up transitions. Many other more complex ionizations are predicted across the energy range encompassed in this study, for which the molecular orbital model of ionization can be said to have effectively broken down. Although these ionizations are mainly of very low probability, their increasing density with increasing energy (that can be identified from the black baseline markings in Figure 2) contributes a small quasi-continuous spectral background in the inner valence region.

### 4.3 Ionization Energies of the HOMO to HOMO-2 Orbitals

#### 4.3.1 Vertical Ionization Energies

As outlined in the Introduction, we have a particular interest in understanding the contribution of the first three cation states, formed by ionization of the oxygen lone-pair orbitals, to the first two apparent band structures in the photoelectron spectrum (PES). Figure 4 shows the first two bands at high resolution. To date assignment has largely been attempted by calculation of ionization energies, but the older literature mostly uses rather small basis sets and rather elementary model chemistries, as judged by current day standards. In this work we have performed ionization energy calculations using a hierarchy of modern levels of theory with some key results for the HOMO–HOMO-2 ionizations summarized in Table 4. For comparison purposes, these use the same triple-ζ cc-pVTZ basis and the staggered molecular geometry. More complete results, comparing augmented and non-augmented cc-pVTZ bases and the staggered and eclipsed geometry forms may be found in Tables S1–S3 (Supporting Information). From the first line of Table 4, it is immediately seen that the simple use of cc-pVTZ canonical orbital energies via Koopmans theorem (KT) suggests ionization energies that are more than 1 eV higher than the experimental bands.

**Figure 4:**
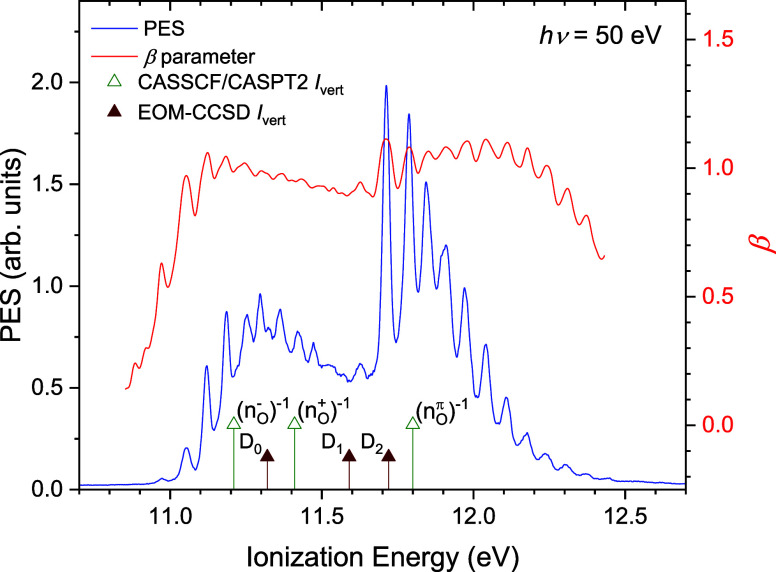
Nitromethane pseudomagic-angle (*I*_par_ + 2*I*_perp_) valence PES and β anisotropy parameter recorded at a photon energy of 50 eV. The positions of the calculated CASSCF-CASPT2/ANO-L-VDZP and EOM-IP-CCSD/cc-pVTZ vertical ionization energies (see Table 4) are marked along the bottom axis.

**Table 4:** Calculated Vertical Ionization Energies, *I*_vert_, in eV, for the First Three Cationic States of Nitromethane^a^

	1st state		2nd state		3rd state	
method^b^	orbital	*I*_vert_	orbital	*I*_vert_	orbital	*I*_vert_
KT^c^	16: *n*_O_^π^	12.38	15: *n*_O_^+^	13.46	14: *n*_O_^–^	13.48
OVGF^d^	16: *n*_O_^π^	11.56 (0.89)	14: *n*_O_^–^	11.60 (0.89)	15: *n*_O_^+^	11.68 (0.90)
ADC(3[4+])^d^	16: *n*_O_^π^	11.50 (0.87)	14: *n*_O_^–^	11.77 (0.87)	15: *n*_O_^+^	11.96 (0.89)
EOM-IP-CCSD^e^	14: *n*_O_^–^	11.32 (0.955)	15: *n*_O_^+^	11.59 (0.957)	16: *n*_O_^π^	11.72 (0.960)
EOM-IP-CCSD/CBS(∞)^f^	14: *n*_O_^–^	11.62	15: *n*_O_^+^	11.90	16: *n*_O_^π^	12.02
RMS-CASPT2^g^	14: *n*_O_^–^	11.21	15: *n*_O_^+^	11.41	16: *n*_O_^π^	11.80

a The cation states are identified by specifying the orbital identity of the single hole configuration.

b Calculations made at the staggered MP2/cc-pVTZ optimized geometry with symmetry disabled and using the cc-pVTZ basis (unless otherwise stated).

c Koopmans theorem, i.e., negative of the HF canonical molecular orbital energy.

d Numbers in parentheses following the *I*_vert_ values are the corresponding pole strengths.

e Numbers in parentheses following the *I*_vert_ values are the amplitudes for the indicated principal ionization (orbital hole).

f Complete Basis Set (CBS) extrapolation of EOM-IP-CCSD/aug-cc-pV*n*Z (*n* = D,T,Q) calculations to *n* = ∞.

g Calculations made at the RMS-CASPT2­(14,11)/ANO-L-VDZP level of theory on top of its optimized staggered geometry without imposing symmetry constraints.

A major improvement is obtained by introducing some treatment for electron correlation. Both the OVGF^17,32^ and IP-ADC­(3­[4+])^35–37,47,48^ methods treat one-hole (1*h*) ionization processes through third-order many-body perturbation theory but, as already seen, ADC(3) variants additionally consider configuration interaction between 1*h* and two-hole-one-particle (2*h*1*p*) cationic states. From Table 4 it can be seen that both bring about a similar significant downward revision of the estimated ionization energies that now fall within the 11–12.4 eV region of the experimental PES bands (Figure 4). It is notable, however, that the second and third ionization energies reverse the ordering of the 15^–1^ and 14^–1^ canonical orbitals.

A next level of treatment explicitly incorporates electron correlation using coupled cluster theory using single and double excitations (CCSD) from the reference determinant. Such EOM-IP-CCSD calculations^38–41^ for the ionization energy offer the prospect of improved accuracy as long as the ionization is well-described as a single-electron transition. The ADC(3) calculations discussed above provide such reassurance that these first three orbital ionizations are predominantly one-electron processes, and the EOM-IP-CCSD/cc-pVTZ results are included in Table 4. The vertical ionization energies predicted for the three O lone-pair derived outer orbitals are further reduced, but the energetic order is now completely reversed from that of the HF canonical orbitals with the HOMO (16) appearing as the more tightly bound of the three. The EOM-IP-CCSD amplitudes (Table 4) do, however, corroborate the strong 1*h* character of these three ionizations.

Tables S1–S3 provide a comparison of the results obtained for the eclipsed conformation by OVGF, ADC­(3­[4+]), and EOM-IP-CCSD methods, respectively. For a given method and basis set, the staggered and eclipsed conformer results are negligibly different throughout the outer valence region. Basis size differences have, however, been observed to be far larger in the course of this work. For example, adding diffuse functions to the cc-pVTZ basis causes a rise in the calculated ionization energies on the order of 0.1–0.2 eV as shown in Tables S1–S3.

A systematic investigation using augmented and nonaugmented members of Dunning’s family of correlation-consistent basis sets, (aug-)­cc-pV*n*Z (*n* = D,T,Q),^45^ was performed with the EOM-IP-CCSD method and the results, extrapolated to the CBS limit,^46^ are included in Table 4. The full results for each individual basis can be found in Table S4 (which offers further evidence of the size-dependent results with finite basis sets). It is noted that the individually extrapolated series for augmented and non-augmented bases converge to effectively the same CBS limits; however, if using just a given member of the D,T,Q family, the augmented version with added diffuse functions evidently gives closer agreement with the best estimate CBS result.

All of the preceding methods are based upon the use of a single reference determinant. For example, the EOM-IP-CCSD method here employs a ground state CCSD calculation to generate the initial single reference wave function. Examination of the so-called T1-diagnostic^69^ for the ground state nitromethane CCSD calculation reveals a value approaching the cusp from where it would be considered an indicator of the desirability of a multideterminant reference function. An early investigation of the ground state of nitromethane, albeit at the minimal basis level,^70^ concluded that a small multiconfiguration configuration interaction was required to reproduce adequately the singlet–triplet energies of this molecule. Hence, to explore whether further refinement of the calculated ionization energies may require a more complete configuration interaction treatment for the reference state, we include a multireference CASSCF-CASPT2 calculation^57,63,64^ (described in Section 3.5) with an atomic natural orbital, large double-ζ basis (ANO-L-VDZP) in the summary given in Table 4.

All of the methods in Table 4 that go beyond the KT result and employ some treatment for electron correlation show a reordering of the cation state energies from that which follows simply from the canonical orbital energy ordering. This clearly has implications for any assignment of the PES bands and indicates an issue for the older literature where attempts to assign the photoelectron peaks were based, either implicitly or explicitly, on the use of Koopmans theorem alone. In particular, those methods that would seem to offer the more complete treatment of electron correlation, EOM-IP-CCSD and CASSCF-CASPT2, both show the energetic ordering (*n*_O_^–^)^−1^ < (*n*_O_^+^)^−1^ < (*n*_O_^π^)^−1^ to be the reverse of the simple KT ordering of (*n*_O_^π^)^−1^ < (*n*_O_^+^)^−1^ < (*n*_O_^–^)^−1^. (We also note in passing that DFT calculations for these three orbitals show a reversed order compared with the HF canonical order. This is not necessarily surprising as Koopmans theorem does not strictly apply for DFT due to different approximations in the exchange–correlation functionals.)

The EOM-IP-CCSD/cc-pVTZ and CASSCF-CASPT2/ANO-L-VDZP vertical ionization energies are marked below the experimental PES in Figure 4. While the theory-experiment agreement looks convincing, there is nevertheless some ambiguity when trying to assign specific experimental structure to the individual ionic states as, given the noted basis size-dependence, the absolute values returned by these two specific calculations may offer fortuitously good agreement. Consequently, further evidence providing some corroboration for the assignments inferred from the comparisons in Figure 4 would be invaluable.

#### 4.3.2 Adiabatic Ionization Energies

All of the calculated values considered in the previous section are for a vertical excitation in which the geometry of the cation state is that of the neutral ground state equilibrium position. The precise location of the vertical ionization energy in an experimental photoelectron band can be somewhat ill-defined, and that certainly is true for the three states associated with the double peaked spectrum observed between 11 and 12.5 eV. As an alternative, ionization thresholds can be estimated as adiabatic ionization energies, and sometimes these may be more readily identified with vibrational origins in the experimental spectra.

Estimates of the adiabatic ionization energies, *I*_adiab_, have been obtained from Δ*E*_SCF_ calculations using *E*_0_ values calculated at the potential minima of the respective states. These are available from the fully optimized DFT (B3LYP and PBE0) geometry calculations performed prior to the vibrational simulations (to be discussed in the next section and as detailed in Section 3.4). Results using the cc-pVTZ and aug-cc-pVTZ bases are listed in Table 5. Note that in all cases, the (*n*_O_^+^)^−1^ state in the eclipsed configuration failed to converge to a stationary minimum. For completeness, we also show in Table 5 the Δ*E*_SCF_ vertical ionization energy estimates obtained at this level of theory using the cation energies fixed at the neutral S_0_ ground state equilibrium geometry. These vertical ionization estimates compare favorably with the more sophisticated computations in Table 4.

**Table 5:** Calculated Δ*E*_SCF_ Ionization Energies (eV) Using Various Density Functionals and Basis Sets

	*I*_adiab_ ^a^ ^,^ ^b^			*I*_vert_ ^b^ ^,^ ^c^		
method	D_0_ (*n*_O_^–^)^−1^	D_1_ (*n*_O_^+^)^−1^	D_2_ (*n*_O_^π^)^−1^	D_0_ (*n*_O_^–^)^−1^	D_1_ (*n*_O_^+^)^−1^	D_2_ (*n*_O_^π^)^−1^
*staggered*						
B3LYP/cc-pVTZ	11.04	11.19	11.68	11.40	11.65	11.86
PBE0/cc-pVTZ	11.0	11.23	11.59	11.39	11.64	11.78
B3LYP/aug-cc-pVTZ	11.13	11.28	11.77	11.48	11.74	11.94
PBE0/aug-cc-pVTZ	11.08	11.30	11.66	11.45	11.72	11.85
RMS-CASPT2/ANO-L-VDZP	10.83		11.66	11.21	11.41	11.80
*eclipsed* ^d^						
B3LYP/cc-pVTZ	11.04		11.67	11.41	11.65	11.86
PBE0/cc-pVTZ	10.98		11.58	11.39	11.64	11.78
B3LYP/aug-cc-pVTZ	11.13		11.75	11.48	11.74	11.94
BPE0/aug-cc-pVTZ	11.05		11.65	11.46	11.72	11.84
RMS-CASPT2/ANO-L-VDZP	10.84	11.27	11.63	11.28	11.49	11.80

a Adiabatic ionization energy Δ*E*_SCF_ = *E*_0_ (D_*n*_) – *E*_0_ (S_0_) evaluated at the potential minima of the respective states.

b Calculation of the excited cation states D_1_ and D_2_ uses MOM to control the electron hole configuration.

c Vertical ionization energy Δ*E*_SCF_ = *E*_0_ (D_*n*_) – *E*_0_ (S0) evaluated at the neutral ground state potential minimum.

d MOM calculations for D_1_ (*n*_O_^+^)^−1^ potential did not converge to a stable minimum in the eclipsed configuration.

### 4.4 Vibrational Structure in the First Two Photoelectron Bands

As will be described below, the first two bands observed in the photoelectron spectrum correspond principally to the D_0_ and D_2_ states, and these will be described first, followed by a discussion of the less evident band, due to the D_1_ state, which lies between them.

#### 4.4.1 CH_3_NO_2_

An overview of the outer valence experimental spectrum, in the energy range of 10.6–18.6 eV, is offered in Figure S2 (Supporting Information) while an expanded region encompassing the lowest energy photoelectron band in CH_3_NO_2_ is plotted in Figure 5a. Several fairly regular vibrational progressions (P1–P4) are observed in the low (11.055–11.250 eV) and the high (11.260–11.421 eV) energy regions of the first band but a discontinuity is evident at ∼11.26 eV. Table S5 contains the ionization energies and tentative assignments for some of the vibrational structure.

**Figure 5:**
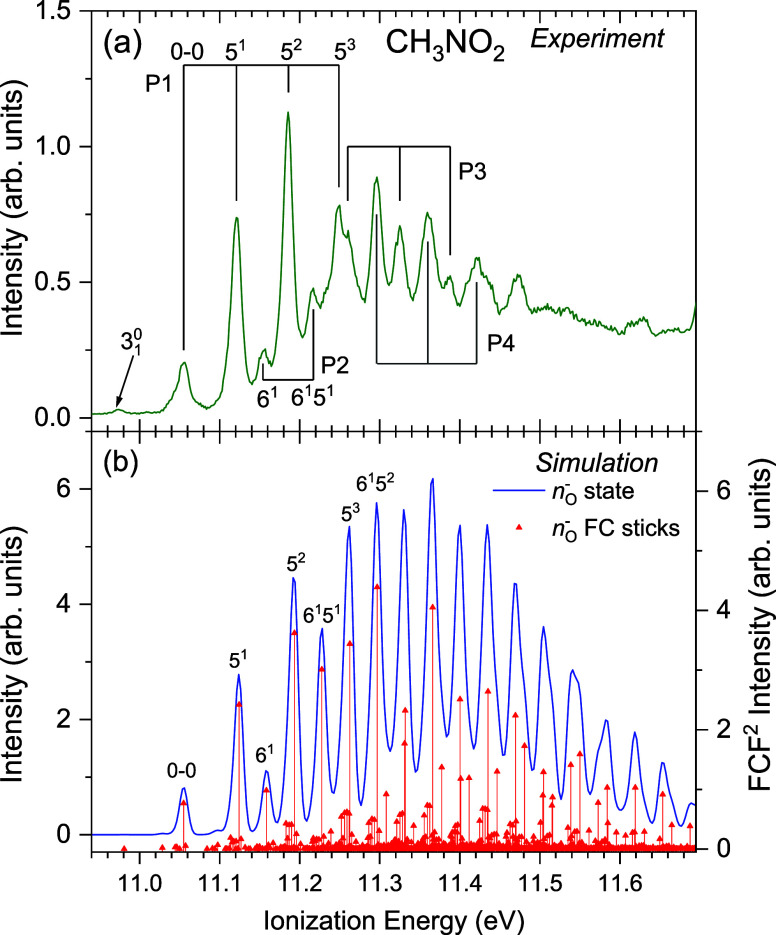
First band region of the experimental CH_3_NO_2_ photoelectron spectrum, recorded at a photon energy of 20 eV, showing identified vibrational progressions and suggested assignments in (a). The ionization energies of the marked peaks in progressions P1–P4 are given in Table S4. A Franck–Condon simulation calculated for the ionized D_0_ (*n*_O_^–^)^−1^ state is shown in (b) both as a stick spectrum, comprising individual transitions and intensities (FC-factors^2^), and as a convolution of these sticks with a Gaussian broadening function of FWHM = 12 meV. The simulations are plotted with the 0–0 vibrationless transition at the position of the identified experimental origin (11.055 eV).

The simulation for the D_0_ (*n*_O_^–^)^−1^ state in the staggered geometry (Figure 5b) indicates that the structure occurring in the low energy region of the first photoelectron band arises from progressions involving modes 5 and 6 either alone or in various combinations with each other. The main progressions, P1 and P2, are assigned as arising from the 5^*n*^ and 5^*n*^6^1^ excitations (where *n* is the number of quanta in the ionized state). Modes 5 and 6 can both be described as C–N bond stretches with synchronized O–N–O scissoring motions but having opposite phasing; for mode 5 the stretch (contraction) and angle closing (opening) motions are in-phase, whereas mode 6 has the stretch (contraction) and angle opening (closure) motions occur in-phase. Note that in the neutral S_0_ state (Table S6), the corresponding vibrational modes are labeled 4 and 5, respectively, due to small frequency shifts of other modes upon ionization.

The peak due to the adiabatic transition (origin) in the D_0_ state is observed at 11.055 eV. Vibrational energies of 66 and 99 meV are derived for modes 5 and 6 in the D_0_ state. These values have been obtained simply as the difference between the ionization energy, quoted with an uncertainty of ±1 meV, of the photoelectron peak due to the 5^1^ or 6^1^ excitation and that of the peak due to the adiabatic transition. All of our reported vibrational energies have been estimated in this manner. The corresponding calculated harmonic vibrational energies are 69 and 103 meV, respectively, in both staggered and eclipsed geometries (see Table S7).

In addition to the main progressions, P1 and P2, our simulation suggests that the peak occurring at 10.973 eV in the experimental spectrum might be a hot-band attributed to the 3_1_^0^ transition. The calculated energy for mode 3 in the S_0_ state is 74 meV in the staggered conformation (75 meV in the eclipsed conformation), whereas the experimental peak occurs at an energy of 82 meV below that due to the origin transition.

Two more progressions, P3 and P4, appear in the high energy region of the first photoelectron band (Figure 5a). In both progressions, the vibrational spacing is only slightly smaller (∼63 meV) than that in P1 and P2, perhaps suggesting that these progressions might involve mode 5. However, the assignments of progressions P3 and P4 are not straightforward because neither the first peak associated with P3 (at 11.260 eV) nor the first peak associated with P4 (at 11.297 eV) appears to arise from a vibrational excitation consistent with the origin occurring at 11.055 eV. Thus, a discontinuity, in terms of both peak intensities and vibrational energies, occurs around 11.26 eV. Finally, the peak at the highest ionization energy in this first band (11.472 eV) does not appear to belong to either P3 or P4.

The simulation for the D_0_ (*n*_O_^–^)^−1^ state (Figure 5b) provides a very satisfactory interpretation of the vibrational structure appearing in the experimental spectrum up to an energy of 11.25 eV but at higher energies the predicted structure is incompatible with the measurement. Notably, the simulation provides no hint of the discontinuity observed at around 11.26 eV.

The experimental spectrum encompassing the second photoelectron band of CH_3_NO_2_ is plotted in Figure 6a, and the simulation for the D_2_ state in a staggered geometry is shown in Figure 6b. Two vibrational progressions (P5 and P6) appear in the experimental spectrum, and the ionization energies are given in Table S5. Our simulation indicates that P5 involves excitation of mode 5. The experimentally derived energy for this vibrational mode in the staggered D_2_ state is 75 meV, compared to the calculated values of 74 (77) meV (where the second value in parentheses is for the eclipsed conformer, Table S9). The experimental peak at 11.712 eV may consequently be assigned to the D_2_ vibrationless origin. At energies between 11.95 and 12.40 eV, the experimental spectrum contains a fairly regular progression with a vibrational spacing of ∼67 meV. This spacing is similar to the 67 (65) meV calculated for mode 4 (Table S9), although this apparent consistency may be fortuitous.

**Figure 6:**
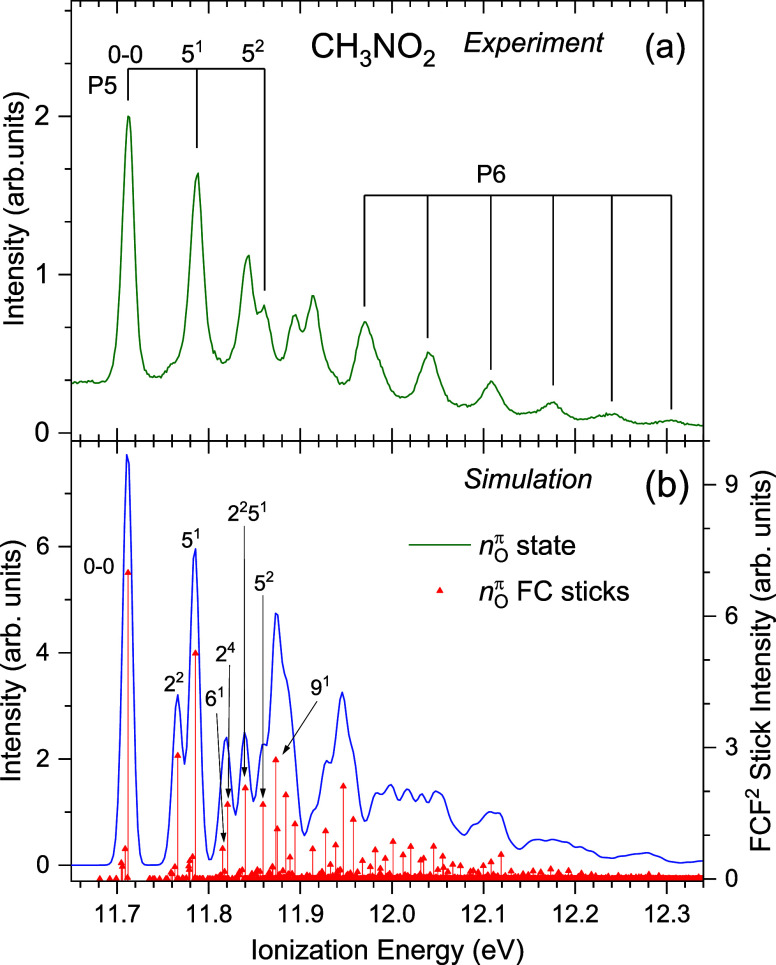
CH_3_NO_2_ experimental photoelectron spectrum ((a), recorded at a photon energy of 20 eV) showing the second band region with the identified P5 and P6 progressions marked and with suggested assignments for the members of the former. Ionization energies for these experimental progressions may be found in Table S4. A Franck–Condon simulation using harmonic frequencies calculated for the D_2_ (*n*_O_^π^)^−1^ ionization is presented in (b). The 0–0 transition in the simulation is set as 11.712 eV to match the experimentally determined band origin. Other details are as in Figure 5.

Two doublets are observed at energies around 11.85 and 11.90 eV (Figure 6a). The peak appearing at 11.843 eV in the experimental spectrum is relatively intense, but a corresponding feature is not predicted in the D_2_ simulation. The higher energy doublet has peaks at 11.894 and 11.915 eV, neither of which appear to form part of either P5 or P6. The structure in this region seems strongly perturbed, with an apparent discontinuity around 11.9 eV.

#### 4.4.2 CD_3_NO_2_

Figures 7 and 8 show the experimental spectra encompassing the two lowest energy bands in CD_3_NO_2_ and the simulations for the D_0_ (*n*_O_^–^)^−1^ and D_2_ (*n*_O_^π^)^−1^ states in the staggered geometry. As expected, the simulations for the (*n*_O_^–^)^−1^ state (Figure 7b) predict that the regular vibrational structure (P7 and P8) observed near the beginning of the first photoelectron band (Figure 7a) should be attributed to progressions involving (*n*_O_^–^)^−1^ modes 5 and 6. The experimentally derived energies for modes 5 and 6 are 65 and 98 meV, compared to the calculated values for the staggered (eclipsed) conformations of 67 (67) and 101 (100) meV (Table S7), respectively. The origin occurs at 11.059 eV and two weak hot-bands appear at 10.983 and 11.016 eV possibly due to the 3_1_^0^ and 2_2_^2^ transitions, respectively.

**Figure 7:**
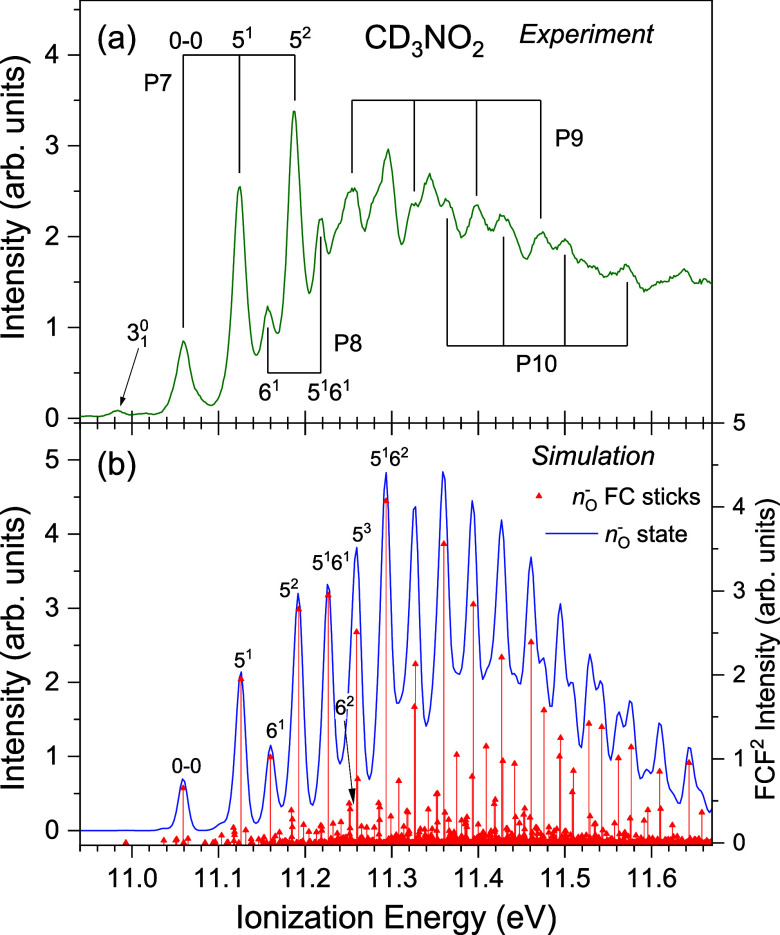
First photoelectron band region of CD_3_NO_2_, recorded at a photon energy of 23 eV. (a) shows the experimental spectrum with four identified progressions (P7–P10) marked and suggested assignments for P7 and P8. The ionization energies of the marked peaks in these progressions are given in Table S4. (b) shows a Franck–Condon simulation for the D_0_ (*n*_O_^–^)^−1^ ionization as both sticks, showing the calculated energy and intensity of individual transitions, and as a smoothed spectrum after convolution with a FWHM = 12 meV Gaussian broadening function. The simulated spectrum is plotted with a 11.059 eV offset to match the 0–0 transition energy with the experimental band origin.

**Figure 8:**
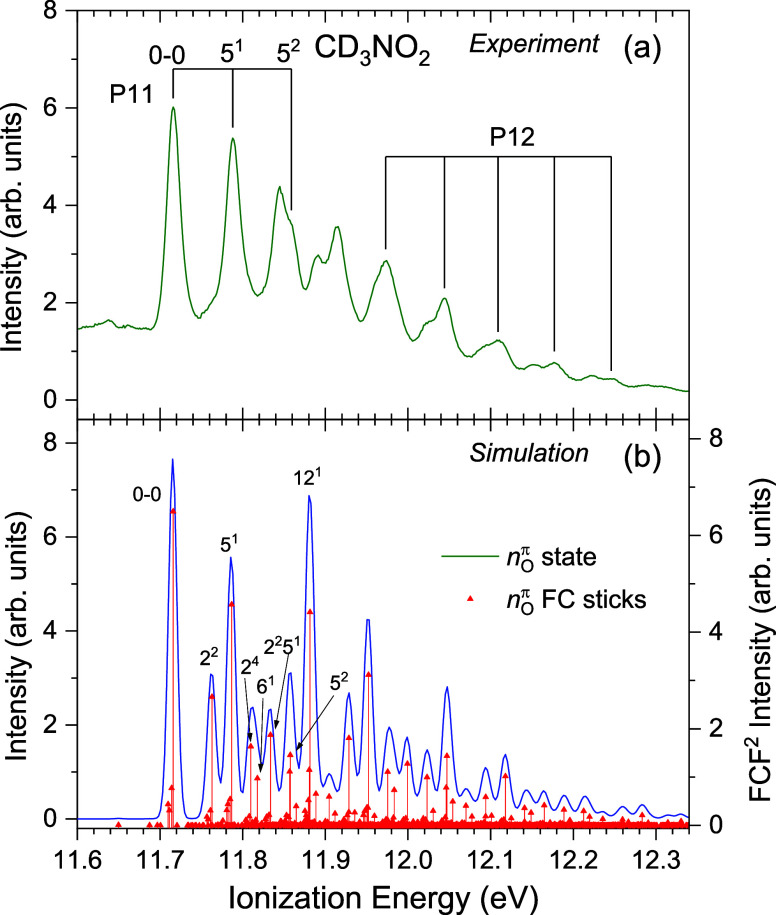
Second photoelectron band region of CD_3_NO_2_, recorded at a photon energy of 23 eV. (a) shows the experimental spectrum with two identified progressions, P11 and P12, and suggested assignments for the former. The ionization energies of these progressions are given in Table S4. (b) shows a Franck–Condon simulation for the D_2_ (*n*_O_^π^)^−1^ ionization as both a stick spectrum and a convolution of these with a 12 meV FWHM Gaussian broadening function. The simulations are plotted with a 11.716 eV offset to match the position of the vibrationless 0–0 transition with the experimentally determined band origin.

A discontinuity in the vibrational structure occurs around 11.25 eV and the assignments of the peaks lying at higher energies remain uncertain. Short progressions (P9 and P10), with vibrational separations of ∼72 meV, are discernible, but unlike those in CH_3_NO_2_, these do not appear to form extended progressions. Whereas in CH_3_NO_2_ P3 and P4 have vibrational spacings that are similar to those in P1 and P2, the experimental vibrational spacings above 11.25 eV in the first CD_3_NO_2_ photoelectron band are ∼72 meV, compared to a mode 5 spacing of 65 meV identified near the beginning of the band. It is of interest to note that although the vibrational energies vary as expected upon deuteration, the structures observed in the regions around the discontinuities in CH_3_NO_2_ and CD_3_NO_2_ are similar, in terms of both profile and energy.

For the second photoelectron band in CD_3_NO_2_ (Figure 8) a short progression (P11) in mode 5 occurs near threshold, where the experimentally derived energy of 72 meV may be compared to the calculated value of 71 (73) meV for the (*n*_O_^π^)^−1^ state (Table S9). Two doublets appear in the experimental spectra around 11.85 and 11.90 eV, which are not evident in the simulation. Toward the high-energy end of the second photoelectron band there appears to be an irregular progression (P12) with a spacing of ∼64–72 meV. This progression is not predicted in the simulation.

### 4.5 The D_1_ (*n*_O_^+^)^−1^ State

While the preceding comparison of the adiabatic normal mode vibrational simulations with the experimental data allows a rather confident assignment of at least the low energy regions of the first two PES bands to, respectively, the D_0_ (*n*_O_^–^)^−1^ and D_2_ (*n*_O_^π^)^−1^ states, it remains to adduce any evidence for the presence of the D_1_ (*n*_O_^+^)^−1^ state. The irregularity in the D_0_ region of the CH_3_NO_2_ PES, discussed above, starts at 11.26 eV, but further irregularities are observed, starting from 11.49 eV and extending up to just below the D_2_ state origin at 11.712 eV. Initially the distinct, sharp peaks cease and are replaced by broader, less distinct weak fluctuations at 11.53 and 11.57 eV. Then, at 11.63 eV, a new distinct sharp peak emerges that seems unconnected with the other well-defined narrow structures. This behavior is quite reproducible as may be seen by comparing the PES measured at *h*ν = 50 eV (Figure 4) with those measured at *h*ν = 30 and 40 eV (Figure 9). Some related structuring appears in the CD_3_NO_2_ spectra between 11.59 and 11.70 eV.

**Figure 9:**
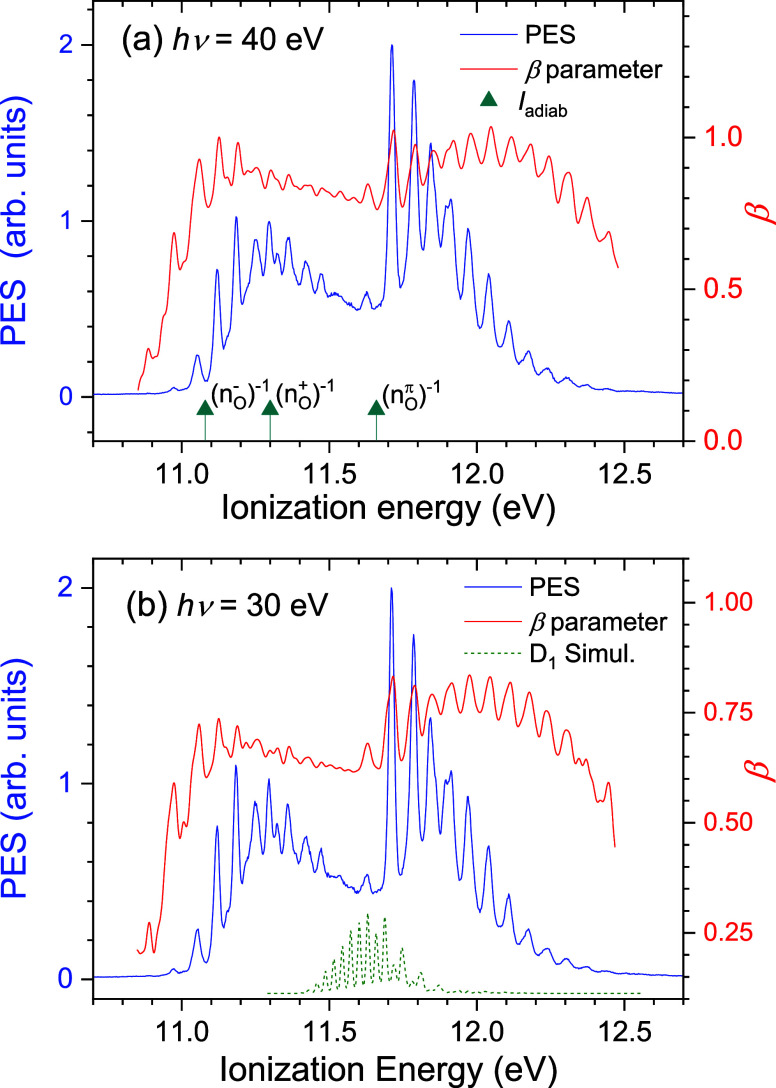
CH_3_NO_2_ pseudomagic-angle (*I*_par_ + 2*I*_perp_) valence PES and β anisotropy parameters recorded with (a) 40 eV and (b) 30 eV photon energies. (a) has the calculated BPE0/MOM/aug-cc-pVTZ adiabatic ionization energy estimates marked, while (b) shows the simulated D_1_ vibrational spectrum with its origin speculatively placed at 11.4 eV.

Although there is some variance in the vertical ionization energies obtained by the coupled-cluster and CASSCF/CASPT2 methods (Table 4), these correlated methods are consistent in placing the (*n*_O_^+^)^−1^ ionization between those of the (*n*_O_^–^)^−1^ and (*n*_O_^π^)^−1^ ionizations and in the vicinity of the spectral features just discussed. Similar predictions are obtained from the less sophisticated Δ*E*_SCF_ DFT/MOM calculations in Table 5. It may then be tentatively suggested that the distinct 11.63 eV PES peak might be evidence of the as yet undetected D_1_ state.

Table 6 shows calculated equilibrium geometries for the S_0_, D_0_, D_1_, and D_2_ states of CH_3_NO_2_. The biggest geometry change is noted for the D_1_ (*n*_O_^+^)^−1^ state, with significantly greater *r*_CN_ and ∠ONO values. These geometry changes can be rationalized because the *n*_O_^+^ orbital has some C–N σ-bonding character (see Figure 1), causing an increase in the C–N separation on ionization, which then also permits the O–N–O to relax toward a linear configuration. As a consequence of these significant geometry changes, the D_1_ origin transition is correspondingly relatively very weak and the simulated Franck–Condon envelope for the S_0_–D_1_ transition (shown in Figure 10) is skewed differently and has a narrower central region of peak intensity (FWHM ∼0.2 eV) compared to the corresponding S_0_–D_0_ and S_0_–D_2_ simulated spectra (Figures 5 and 6). There is, however, a long weak tail of transitions dispersed toward higher energies. The more prominent structure arises from progressions involving modes 2 and 3, and, at higher energies, also mode 5, with calculated energies for these modes of 28.8, 29.2, and 85.8 meV, respectively (Table S8).

**Table 6:** Optimized Geometries of the Staggered and Eclipsed CH_3_NO_2_ S_0_ and D_0_–D_2_ Cation States and Calculated Positions of the D_0_/D_1_ and D_1_/D_2_ MECI

	staggered			eclipsed		
	*R*(C–N)	*R*(N–O)	∠ O–N–O	*R*(C–N)	*R*(N–O)^a^	∠ O–N–O
B3LYP/cc-pVTZ^b^						
S_0_	1.498 Å	1.218 Å	125.8°	1.499 Å	1.218 Å	125.8°
D_0_ (*n*_O_^–^)^−1^	1.467 Å	1.227 Å	110.6°	1.467 Å	1.224 Å	110.6°
D_1_ (*n*_O_^+^)^−1^	1.907 Å	1.177 Å	140.8°			
D_2_ (*n*_O_^π^)^−1^	1.486 Å	1.248 Å	117.6°	1.479 Å	1.196 Å 1.316 Å	117.8°
CASSCF-CASPT2/ANO-L-VDZP^c^						
S_0_	1.505 Å	1.239 Å	125.8°	1.506 Å	1.239 Å	125.8°
D_0_ (*n*_O_^–^)^−1^	1.497 Å	1.251 Å	110.5°	1.497 Å	1.251 Å	110.5°
D_1_ (*n*_O_^+^)^−1^				1.900 Å	1.207 Å	139.1°
D_2_ (*n*_O_^π^)^−1^	1.510 Å	1.264 Å	117.8°	1.509 Å	1.265 Å	117.8°
MECI^d^ (*n*_O_^–^/*n*_O_^+^)	1.578 Å	1.243 Å	126.9°	1.580 Å	1.243 Å	126.8°
MECI^e^ (*n*_O_^π^/*n*_O_^+^)	1.511 Å	1.265 Å	117.9°	1.514 Å	1.254 Å 1.276 Å	118.5°
experimental^f^						
S_0_	1.489 Å	1.224 Å	125.3°			

a The O atoms are not symmetry equivalent in the eclipsed conformation. Where the two *R*(NO) distances differ significantly both are given.

b The (*n*_O_^+^)^−1^ geometry optimizations by the B3LYP (MOM) method did not converge for the eclipsed conformation.

c CASSCF/CASPT2 (*n*_O_^+^)^−1^ geometry optimizations for the staggered conformation similarly failed to converge.

d D_0_/D_1_ MECI energy 11.21 eV.

e D_1_/D_2_ MECI energy 11.63 eV.

f Experimental parameters for the neutral ground state taken from ref 81.

**Figure 10:**
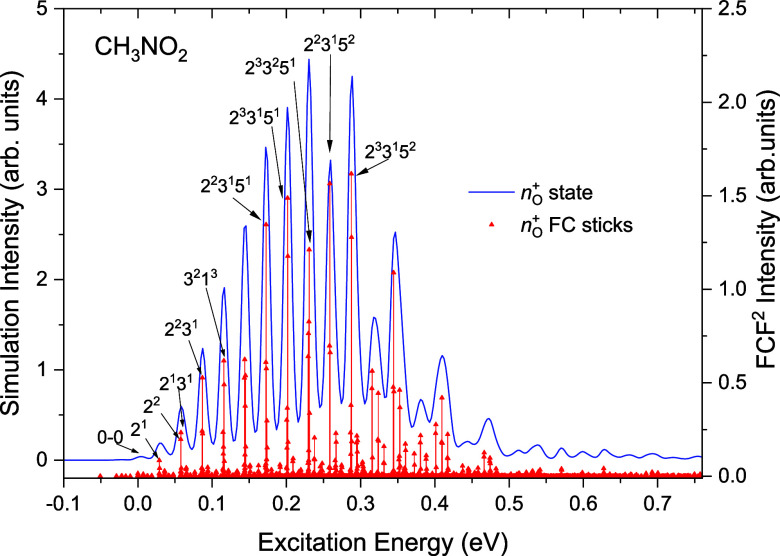
Simulated photoelectron spectrum of the D_1_ (*n*_O_^+^)^−1^ state of CH_3_NO_2_. The simulations have been plotted as stick spectra, using calculated transition energies and intensities, and as a convolution, using a Gaussian broadening function of 12 meV (FWHM). Assignments are marked for some prominent low-energy transitions.

The simulation for the D_1_ state of CH_3_NO_2_ in the staggered geometry is shown again in Figure 9b, speculatively placed with its origin at 11.4 eV and thus with its intensity peaking at ∼11.6 eV—broadly consistent (to within the likely accuracy of the calculations) with, respectively, the calculated adiabatic and vertical ionization energies appearing in Tables 5 and 4. Because of the poor geometric overlap, the Franck–Condon intensity for the D_1_ band, integrated over all energetically accessible levels, is less than half that of the adjacent states. Furthermore, for photon energies below ∼40 eV the calculated electronic ionization cross sections are the smallest for the D_1_ state (see Figure 11). With both the photoionization cross section and Franck–Condon intensity being less than those of the adjacent bands, a D_1_ band may plausibly be a relatively minor contributor to the overall photoelectron spectrum. Nevertheless, it is clear that the structured D_1_ simulation features cannot be uniquely identified in the experimental spectrum. This may be attributable to spectral congestion, exacerbated by an intrinsically weak D_1_ signal, or because of vibronic interaction and broadening through interaction with both nearby states.

**Figure 11:**
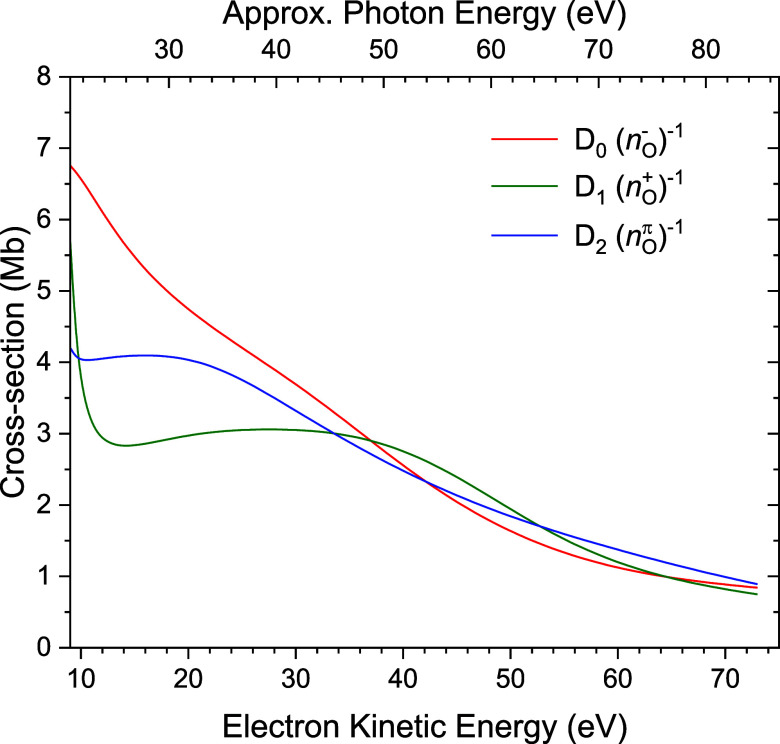
Theoretical (CMS-Xα) calculations of the D_0_, D_1_, D_2_ photoionization cross sections. For convenience, an approximate indication of the corresponding experimental photon energy is given along the top axis. This approximate energy has been obtained by addition of the mean ionization energy (11.8 eV) to the electron kinetic energy.

### 4.6 Photoelectron Angular Distributions

Experimental photoelectron anisotropy parameters (β), derived using eq 2, are included in the plots of photoelectron spectra recorded with photon energies of 50 eV (Figure 4), 40 eV, and 30 eV (Figure 9). These β parameter spectra largely replicate the vibrational structure evident in the PES. Indeed, rather weak features in the PES, including here hot-bands, may be more prominent in the β spectra. In general terms, values of β are larger for the second band and increase with increasing photon energy. For either band, at a given photon energy, the β parameters peak near its origin and then gently reduce across the band. Such a decline may be attributable to simple energetic effects as, at a fixed photon energy, the electron momentum (and hence also typical β values) will decrease as the level of vibrational excitation (ionization energy) of a state increases. One might also consider purely vibrational effects, or vibronic coupling between the ionic states, that could lead to changes in the observed angular distributions. Of course, as long as the Born–Oppenheimer and Franck–Condon approximations are valid, β would be expected to be independent of the level of vibrational excitation. However, previous experimental and theoretical studies on *cis*- and *trans*-dichloroethene^71–73^ showed a theoretically predicted^74,75^ variation in the β-parameter across a photoelectron band when the ionic state was affected by vibronic coupling.

In Figure 12 we plot the calculated CMS-Xα β parameters for the *n*_O_^–^, *n*_O_^+^, and *n*_O_^π^ ionizations. Below ∼10 eV kinetic energy these calculations show evidence of shape resonant behavior—albeit almost certainly exaggerated in this level of calculation where a fixed nuclear geometry is employed. However, above 10 eV kinetic energy, i.e., in the range spanned by our experimental data, the β parameters for all three states display a smoothly varying, gradual increase. Notably, the β values for the D_2_ (*n*_O_^π^)^−1^ state consistently exceed those for the D_0_ (*n*_O_^–^)^−1^ state by a small amount. Because these CMS-Xα calculations exclude any treatment of vibration, we choose to compare them with the experimental CH_3_NO_2_ β values recorded at the peak positions of the vibrationless D_0_ and D_2_ origins (identified in Section 4.4.1). These experimental values are plotted as a function of the electron kinetic energy (*h*ν-ionization energy) in Figure 12. While a little lower than the calculated β curves, the experimental values are in good semiquantitative agreement. The experimental values for each state seem to rise in parallel with their corresponding calculated curve and show that β­(D_2_) is greater than β­(D_0_) across the energy range spanned in Figure 12. The angular distribution measurements thus corroborate the assignments of the first PES band as the D_0_ (*n*_O_^–^)^−1^ state and the second PES band as the D_2_ (*n*_O_^π^)^−1^ state, as first deduced in Section 4.4.1 from analysis of the PES vibrational structure.

**Figure 12:**
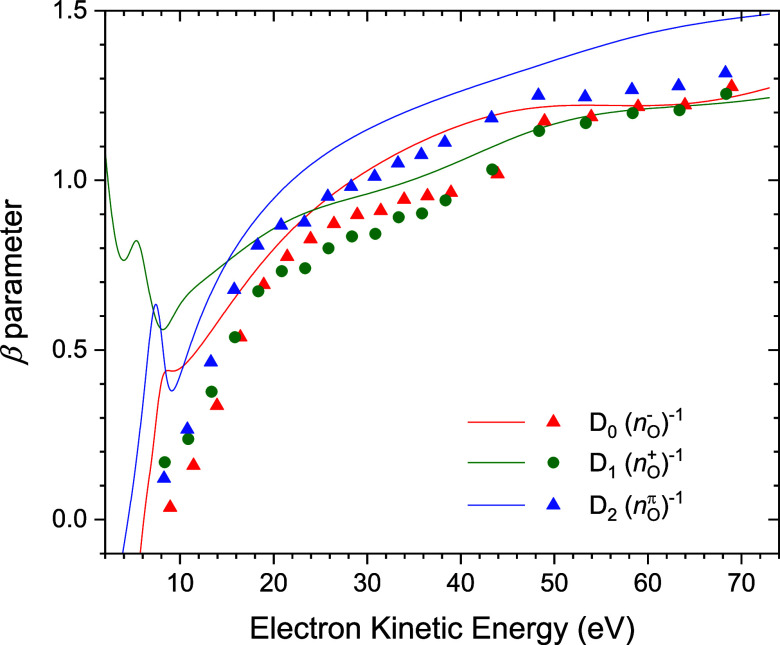
Theoretical (CMS-Xα) CH_3_NO_2_ anisotropy parameters, β, for the D_0_, D_1_, D_2_ states are plotted as solid curves—see graph legend. The experimental β values (solid symbols) measured at the peaks assigned as the D_0_ and D_2_ 0–0 origins and the small peak (ionization energy = 11.63 eV) that has been tentatively identified as belonging to the D_1_ state are plotted for comparison.

Finally, we consider the small yet distinct PES peak noted at 11.63 eV and tentatively suggested to be associated with the D_1_ (*n*_O_^+^)^−1^ state (see Section 4.5). As may be seen in the individual examples recorded at photon energies of 50 eV (Figure 4), 40 eV, and 30 eV (Figure 9) the corresponding β spectra display equally sharp, definite structure at this point, with values that appear quite distinct from any of the adjacent D_0_ or D_2_ peaks. The full set of experimentally derived β values for the 11.63 eV feature has been plotted versus electron kinetic energy in Figure 12. Here it is seen that although the experimental β­(D_1_) values are initially greater than the experimental β­(D_0_) values in the lowest energy region, from about 20 eV upward they fall below the β­(D_0_) values and attain essentially the same magnitudes above the 50 eV kinetic energy. Once again, this behavior achieves semiquantitative agreement now with the calculated D_1_ (*n*_O_^+^)^−1^ β curve, which can be seen to have a higher value than that calculated for the D_0_ (*n*_O_^–^)^−1^ state near the threshold but to increase less slowly as the kinetic energy increases. Hence, although the 11.63 eV PES feature cannot be unambiguously assigned, the angular distribution measurements reinforce the hypothesis that it is associated with the D_1_ (*n*_O_^+^)^−1^ state.

Previous measurements of the photoelectron angular distribution for CH_3_NO_2_,^2^ although lacking any vibrational resolution, made observations consistent with our present findings. At HeI (21.2 eV) photon energy, the intermediate region (∼11.6 eV) between the two bands showed a small increase in β after passing through the first band. A second measurement with Ne I radiation (16.6 eV) observed that the value of β in the intermediate region exceeded that at the center of either the first or second PES band. Although these measurements were recorded at photon energies below those accessible in the present work, the observed experimental behavior represents a plausible extrapolation from the results we obtained at *h*ν = 20 eV, based upon the experimental and theoretical trends shown in Figure 12. Hence, these authors concluded, as here, that the “missing” third electronic state was located at ionization energies intermediate between the more obvious first and second PES bands.

### 4.7 Nonadiabatic Interaction

The apparent absence in the experimental spectra of clearly delineated vibrational progressions or sequence structures resembling FC predictions made for the D_1_ state, together with the restricted energy ranges over which the simulations for the D_0_ and D_2_ states match the peaks in the first and second photoelectron bands, respectively, suggests that the Born–Oppenheimer approximation, wherein each electronic state is characterized by its own potential energy surface, decoupled from those of other electronic states, is not applicable to the D_0_, D_1_, and D_2_ states of nitromethane. An assumption of the validity of the Born–Oppenheimer approximation is implicit in the use and calculation of Franck–Condon factors for transitions between initial neutral and final ionic states.^76^ Vibronic coupling, namely, the interaction between two or more energetically close-lying states through the nuclear motion, represents a breakdown of the Born–Oppenheimer approximation.^77,78^ The ensuing nonadiabatic effects, which account for the motion of the nuclei on more than one potential energy surface, can lead to changes in the band structure of the photoelectron spectrum. Such changes are evident in the appearance of irregular vibrational separations and peak intensities. Detailed studies of vibronic coupling in the low-lying ionic states of *cis*- ^71,72^ and *trans*-^73^ dichloroethane and imidazole^28,79^ have been published recently.

A qualitative understanding of the vibronic interactions that are likely to be affecting the D_0_, D_1_, and D_2_ ionic states in nitromethane may be gleaned by examining potential energy surface cuts made along the vibrational normal modes. A complete set of such cuts along all of the normal mode coordinates is provided in Figure S3. The cuts for in- and anti-phase *r*_CN_ stretch (contraction) plus ∠ONO close (open) modes call for particular attention and are plotted here in Figure 13. Note that in the ground state neutral these are modes 4 and 5, respectively, but in the cations these motions correspond to modes 5 and 6, respectively. These were the two most important modes identified in the analysis of the PES vibrational structure (Section 4.4. Vibrational Structure in the First Two Photoelectron Bands).

**Figure 13:**
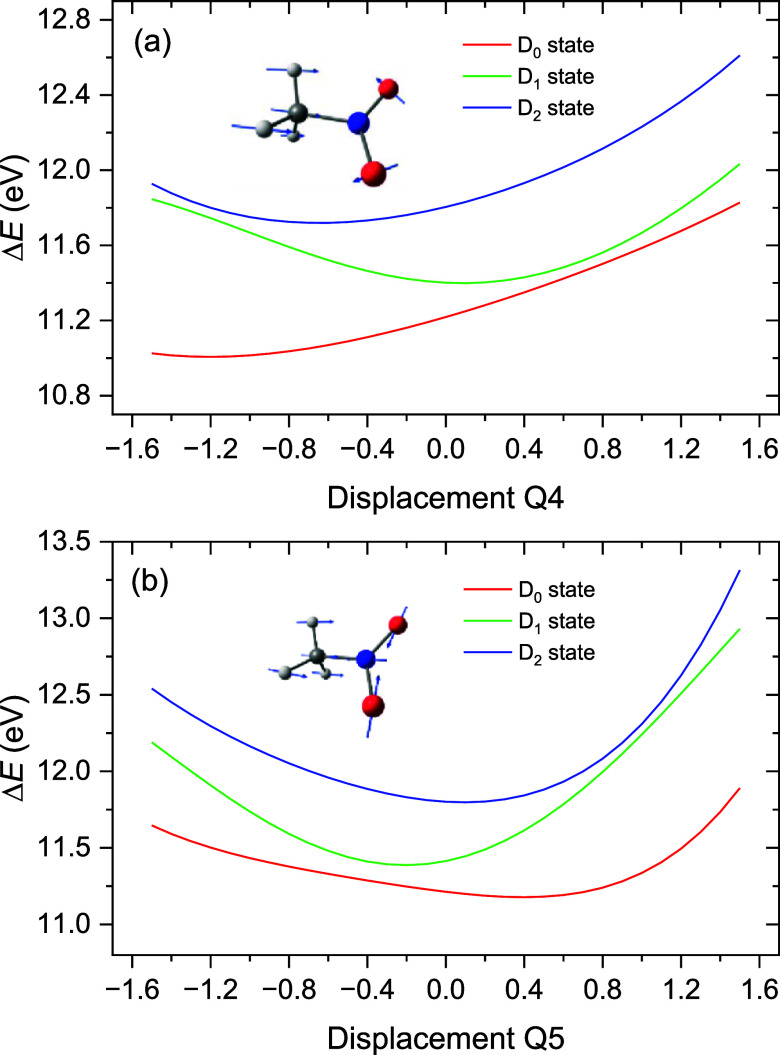
Adiabatic potential energy surface cuts along (a) the in-phase and (b) the antiphase *r*_CN_ stretch (contraction) with ∠ONO close (open) vibrational coordinates obtained from the RMS-CASPT2/ANO-L-VDZP calculations. The zero displacement corresponds to the S_0_ ground state equilibrium geometry. A three-dimensional representation of displacements along both of these coordinates may be found in Figure S6.

Figure 13a shows that the adiabatic potential energy surfaces for the in-phase stretch/scissor motion have an avoided crossing at 11.49 eV, between D_0_ and D_1_, and another avoided crossing at 11.81 eV, between D_1_ and D_2_. Likewise, the energy surfaces for the antiphase motion (Figure 13b) have avoided crossings at 11.41 and 12.19 eV. Thus, the avoided crossings between D_0_ and D_1_ at 11.41/11.49 eV are close to the energy (11.26 eV) of the first vibrational discontinuity in the lowest energy photoelectron band. Similarly, the avoided crossing at 11.81 eV between D_1_ and D_2_ closely matches the energy at which the erratic structure first appears in the second photoelectron band. We may thus strongly suspect that these avoided crossings in the in-phase and antiphase stretch/NO scissoring motion potential cuts are related to the irregularities noted in the cation modes 5 and 6 vibrational progressions.

From the existence of avoided crossings, we may infer the presence of nearby conical intersections, where the adiabatic Born–Oppenheimer model will no longer be valid. The minimum energies and location of the conical intersections corresponding to these avoided crossings were identified using RMS-CASPT2/ANO-L-VDZP calculations and the results are included in Table 6. Here it is seen that the D_0_/D_1_ MECI sits at a C–N distance close to 1.6 Å, while the D_1_/D_2_ MECI is located at a C–N distance of ∼1.5 Å. The latter is very similar to the C–N distance in the neutral S_0_ equilibrium geometry and so will lie close to the *r*_CN_ vertical excitation region in the photoionization (see Table 6). In contrast, the D_0_/D_1_ MECI ONO angle of ∼127° is very similar to the neutral bond angle of 125.8°, while that of D_1_/D_2_ is much reduced to ∼118°.

While MECIs are of inherent interest in computational photochemistry, it has long been recognized that accessibility constitutes a necessary, though not sufficient, requirement for such crossings to strongly dictate reactivity.^80^ To further analyze the accessibility of these CI crossings in the photoelectron spectrum, a more detailed examination of the locus of the D_0_/D_1_ and D_1_/D_2_ seams of intersection with key nuclear degrees of freedom was undertaken. The full results, showing details of the topography and classification of the conical intersections, are presented in Figure S4 with an associated commentary. Briefly summarizing, these show that the seams are relatively insensitive to CH_3_NO_2_ torsion angles and *r*_NO_ distances but that the D_0_/D_1_ seam location, in particular, has a strong mutual dependence on ∠ONO with *r*_CN_.

Figure 14 shows the optimized seams of the conical intersections along the (constrained) C–N distance. Interestingly, the only intersection seam energetically accessible to the first experimental discontinuity at ∼11.26 eV is that of the D_0_/D_1_ interaction, where the predicted energy of 11.21 eV for the MECI lies very close to that of the observed discontinuity. This similarity further supports the proposition that the D_0_/D_1_ crossing is responsible for the first vibrational discontinuity in the photoelectron spectrum.

**Figure 14:**
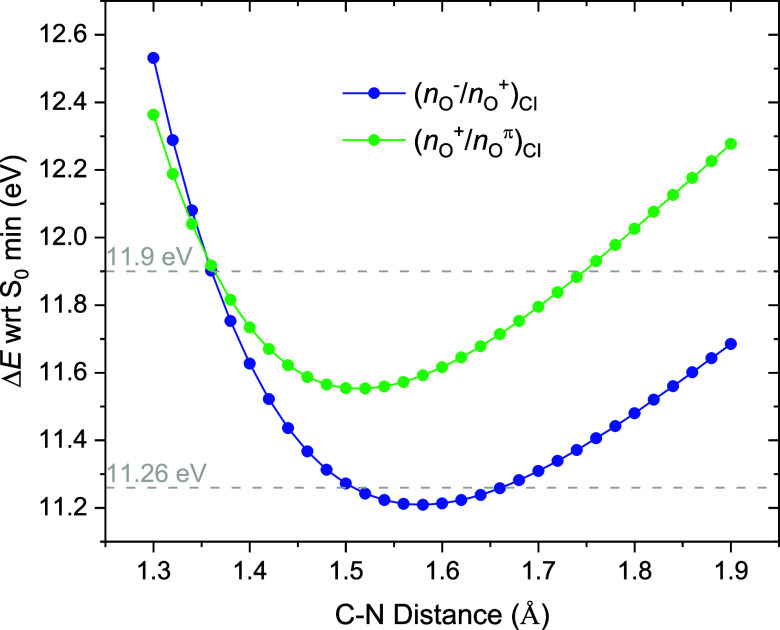
Adiabatic energies (with respect to S_0_ min) of the D_0_/D_1_ ((*n*_O_^–^/*n*_O_^+^)_CI_; in blue) and the D_1_/D_2_ ((*n*_O_^+^/*n*_O_^π^)_CI_; in green) conical intersection seams along the C–N distance at the RMS-CASPT2 level of theory. Gray dashed lines represent the energies at which discontinuities were observed in the vibrational progressions of the experimental bands. Plots showing the locus of the seams along the *r*_CN_, ∠ONO, and O–N–C–H torsion angles are provided in Figure S4.

The vibrational disruption experimentally observed at ∼11.9 eV, on the other hand, occurs at an energy significantly higher than either of the CI minima in Figure 14, but coincidentally, both the D_0_/D_1_ and D_1_/D_2_ seams attain this energy at a decreased C–N distance of ∼1.35 Å. More extensive analysis (Figure S4) shows that accessing this D_1_/D_2_ seam then requires ∠ONO to be less than the S_0_ equilibrium geometry of 126°, while the D_0_/D_1_ seam increases to ∼11.9 eV only when ∠ONO is significantly more open than the S_0_ geometry. On balance, the D_1_/D_2_ crossing appears to require less nuclear rearrangement following photoionization, and we may speculate that this might be primarily responsible for the erratic progressions observed at 11.9 eV.

A 3D graphical overview of the conical intersections coupling the D_0_, D_1_, and D_2_ potential surfaces discussed above may be found in Figure S5.

## 5 Summary

The outer valence shell photoelectron spectrum of CH_3_NO_2_ has been investigated by using linearly polarized synchrotron radiation to record polarization-dependent spectra in the photon energy range 20–80 eV, and some additional spectra of CD_3_NO_2_ have been recorded at low photon energies. Particular attention has been focused on the two lowest energy photoelectron bands that are predicted to arise from ionization of the three outermost orbitals, all of which correspond to lone-pair combinations largely localized on the oxygen atoms. The predicted ionization energies of these orbitals have been shown to be dependent on the level of calculation and the employed basis set. Thus, the results from previous theoretical studies do not allow unambiguous assignments to be made.

More sophisticated calculations, which include a good treatment of electron correlation, such as those used in the present work, predict ionization energies with an ordering of (*n*_O_^–^)^−1^ < (*n*_O_^+^)^−1^ < (*n*_O_^π^)^−1^ These calculated vertical ionization energies fall within the energy range encompassed by the first two photoelectron bands. However, predicted vertical ionization energies are often difficult to identify unambiguously from experimental peak intensities, and this is certainly true for the highly structured bands measured for nitromethane. On the other hand, the origin of an experimental photoelectron band, which corresponds to the adiabatic ionization energy, may sometimes be more readily identified. Here, vibrational assignments assist with such an identification. The experimentally derived adiabatic ionization energies of the first two bands were compared with the corresponding calculated values for the three outermost orbitals, and this has helped clarify the assignments.

Well resolved vibrational structure was observed in the two lowest energy photoelectron bands of CH_3_NO_2_ and CD_3_NO_2_. Close to threshold, in both bands, this structure can be ascribed to regular progressions, but at higher energies, the structure becomes erratic. For both CH_3_NO_2_ and CD_3_NO_2_ the energies at which these irregularities occur in the two bands are similar within the experimental uncertainty. This similarity provides support for the argument that the irregularities involve vibronic coupling between electronic states, since the electronic potentials for the two isotopomers will be the same within the Born–Oppenheimer approximation. Hence the intersections will lie at the same energy, and consequently the irregularities will appear at the same energy.

Franck–Condon simulations for the D_0_ (*n*_O_^–^) and D_2_ (*n*_O_^π^) states have allowed the band origins to be identified and the observed low energy regular structure to be assigned to excitation of specific vibrational modes, thereby corroborating the evidence provided by the calculated ionization energies.

A comparison between the experimentally derived β parameters for the peaks assigned as the origins of the D_0_ and D_2_ states and those obtained from our CMS-Xα calculations provides further support for the predicted ionization energy ordering of (*n*_O_^–^)^−1^ < (*n*_O_^π^)^−1^ In addition, the comparison indicates that a weak but distinct photoelectron peak occurring at an energy below that of the D_2_ (*n*_O_^π^) origin band can tentatively be assigned to the D_1_ (*n*_O_^+^) state, consistent with the calculated ionization energies (*n*_O_^–^)^−1^ < (*n*_O_^π^)^−1^. The theoretical ionization cross sections and the Franck–Condon factors both indicate that the D_1_ (*n*_O_^+^) state ionization may be relatively weak, thereby partially accounting for the weak appearance of this state in the spectrum.

The harmonic vibrational analyses of the progressions in the two lowest energy photoelectron bands have identified energies above which the structure becomes irregular The nonharmonic methyl torsional motion, which can be expected to introduce additional coupling mechanisms, was explicitly neglected. Otherwise, our theoretical explorations suggest that the torsional motion results in very small energy differences that are not expected to change either the state ordering or the expected reactivity (Figure S6). Likewise, the applied harmonic analysis discounts any possible consequences of, e.g., Fermi resonance. Rather, the evidence provided in this study strongly indicates vibronic coupling of the D_0_, D_1_, and D_2_ states in nitromethane.

Theoretical potential energy surface cuts along pertinent vibrational modes show that the energies of the observed vibrational irregularities coincide with predicted avoided crossings between the D_0_ and D_1_ states, and the D_1_ and D_2_ states, thereby suggesting that nonadiabatic interactions in these regions may be affecting the D_0_ and D_2_ photoelectron bands. Such nonadiabatic interactions can result in both a redistribution of spectral intensities and vibrational energies that become modified from those associated with isolated states. It is further implied that any structure associated with the D_1_ state will be affected by vibronic interactions across much of its energy range, thus providing another reason why the band may be both highly irregular and indistinct in the photoelectron spectrum.

Our theoretical modeling of possible vibronic coupling between ionic states is developed by identifying and characterizing extended seams of conical intersections connecting the D_0_/D_1_ and D_1_/D_2_ states. These regions of degeneracy are readily accessible upon photoionization by nuclear motions combining *r*_CN_ stretching and ∠ONO bending oscillations (viz., cation vibrational modes 5 and 6) and so may qualitatively account for the disruption in the vibrational progressions observed experimentally. Unfortunately, a more in-depth theoretical investigation of such coupling, including torsional effects, to yield quantitative estimates is beyond the scope of the present work.

The complete valence shell photoelectron spectrum of nitromethane, measured at a photon energy of 80 eV, exhibits several distinct peaks at ionization energies below ∼20 eV, followed by increasingly broad features in the inner valence region The interpretation of the experimental spectrum has been facilitated by a simulation spectrum obtained by convoluting the pole strengths and corresponding ionization energies obtained from an IP-ADC(3) calculation. This theoretical approach extends beyond the independent electron 1*h* model to include the 2*h*1*p* transitions. The simulation has enabled various shakeup satellite transitions to be identified in the experimental spectrum and has confirmed the importance of electron correlation in the inner valence region.

## Supplementary Material



## Data Availability

The data that support the findings of this study are available upon reasonable request from the authors.

## Notes

Additional computational results including energies and vibrations and potential surface calculations (PDF)
