# Supplementary material for: An Experimental and Theoretical Study of the Valence Shell Electronic Structure of Nitromethane
Source: J Phys Chem A. 2025 Sep 17;129(38):8798–816. doi: 10.1021/acs.jpca.5c04698 (PMC12478869; doi:10.1021/acs.jpca.5c04698)
Supplement: Supplementary file 1 [file jp5c04698_si_001.pdf]

Supporting Information

An Experimental and Theoretical Study of the Valence Shell Electronic Structure of Nitromethane

Ivan Powis[1], Juliana Cuéllar-Zuquin[2], Angelo Giussani[2], Javier Segarra-Martí[2], Bérenger Gans[3], Ugo Jacovella[3], John D Bozek[4], Stephen T Pratt[5], and David M P Holland[6]

[1]*School of Chemistry, The University of Nottingham, University Park, Nottingham NG7 2RD, United Kingdom*

[2]*Instituto de Ciencia Molecular, Universitat de València, C/ Catedrático José Beltrán 2, 46980 Paterna, Valencia, Spain*

[3]*Institut des Sciences Moléculaires d'Orsay, CNRS, Université Paris-Saclay, F-91405 Orsay, France*

[4]*Synchrotron SOLEIL, l'Orme des Merisiers, Départmentale 128, 91190 Saint-Aubin, France*

[5]*Chemical Sciences and Engineering Division, Argonne National Laboratory, Lemont, Illinois 60439, USA*

[6]*ASTeC, Science and Technology Facilities Council, Daresbury Laboratory, Warrington, WA4 2DS, UK*

Figure S1. Active Space Orbitals

CASSSCF orbitals for multiconfigurational calculations (see main text)

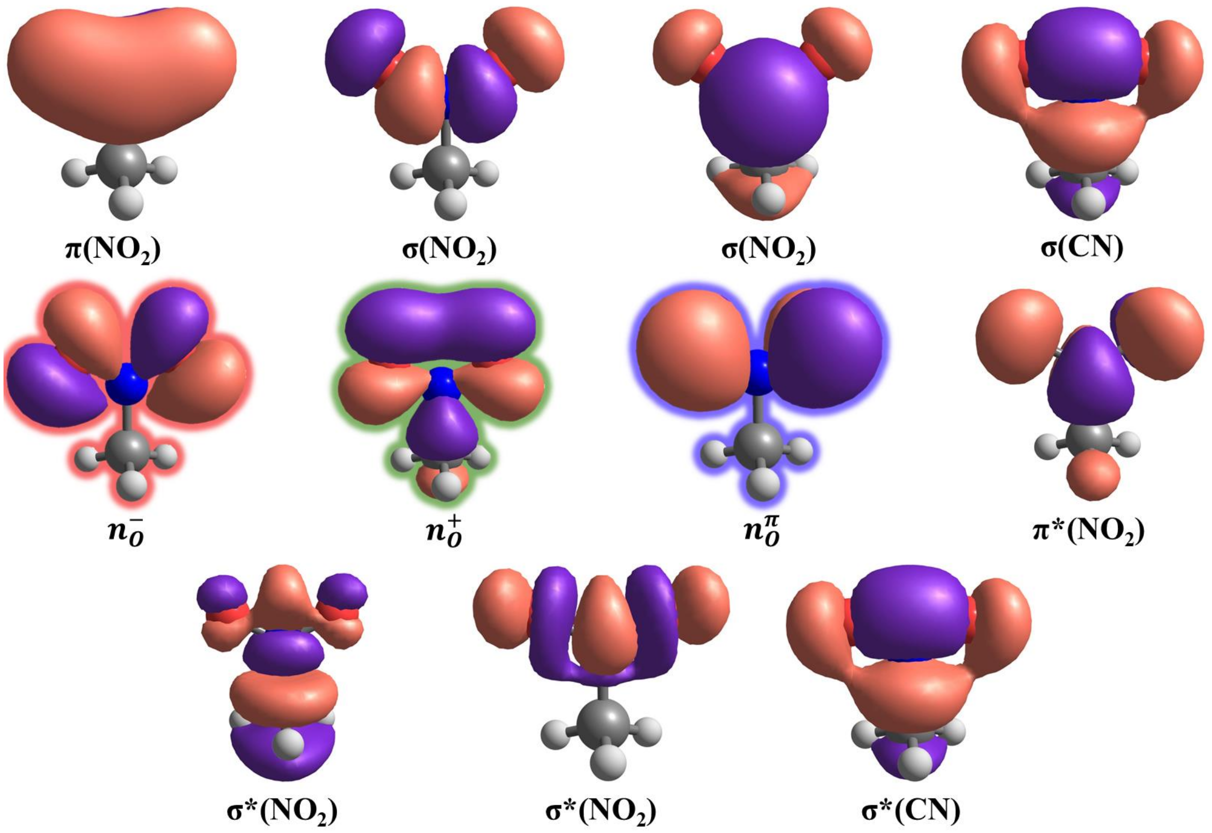

Figure S2. Valence Shell PES

The complete valence shell photoelectron spectrum of CH_3NO_2, recorded at a photon energy of 80 eV, using linearly polarized synchrotron radiation with the electric vector lying parallel to the electron detection axis.

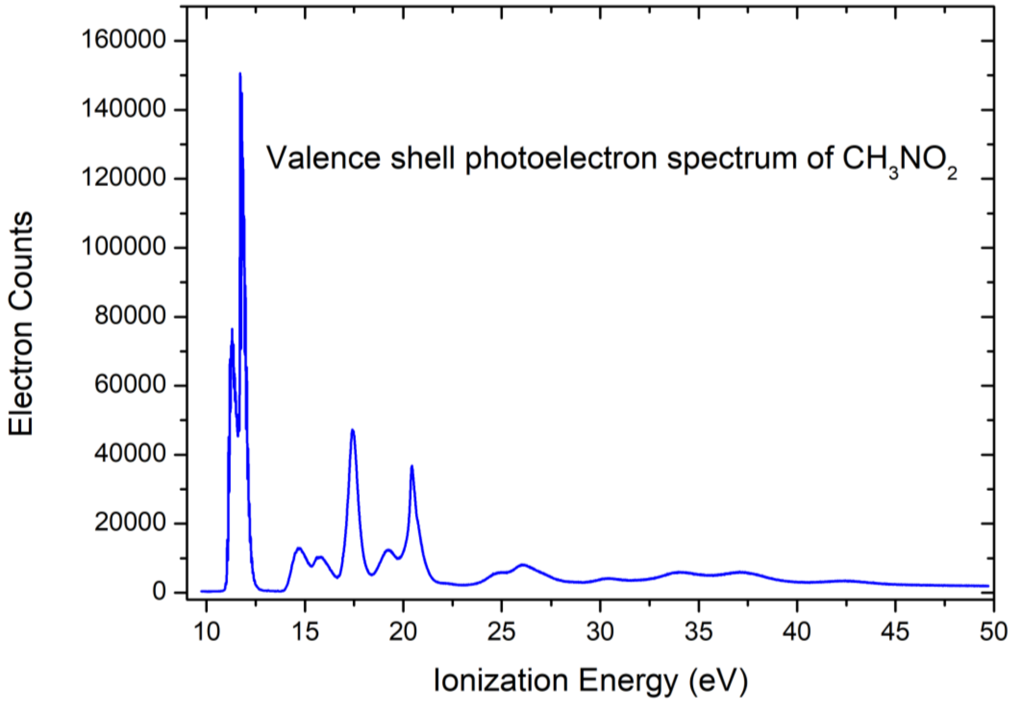

The outer valence region photoelectron spectrum of CH_3NO_2 recorded at a photon energy of 25 eV. Recordings made with linear horizontal and vertical light polarizations have been combined to simulate a "magic angle" spectrum with polarization independent intensities.

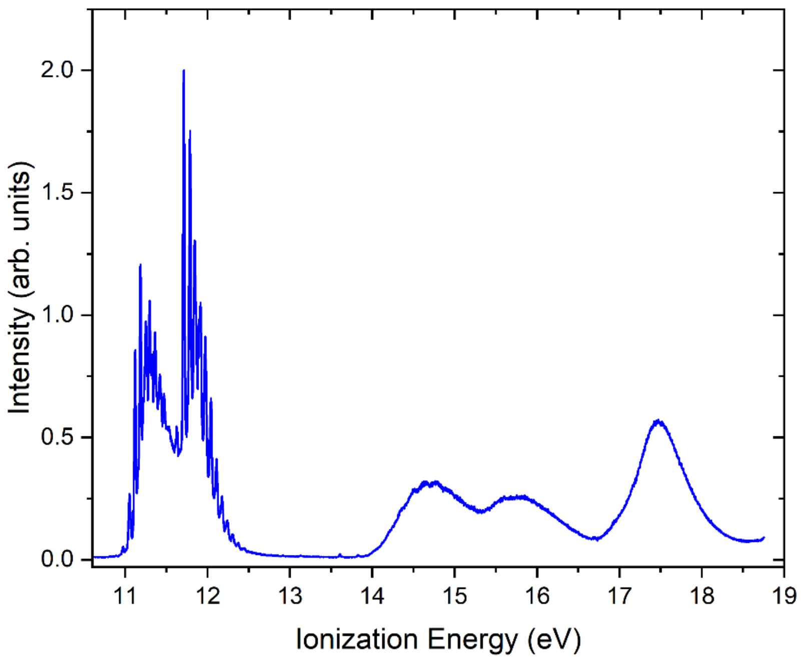

Figure S3. Potential Energy Cuts

Potential energy surface cuts along each S_0 vibrational normal mode. Note particularly that the modes 4 and 5 shown here correspond to the motions of modes 5 and 6 in the cations, due to small shifts in the relative vibrational energies upon ionization.

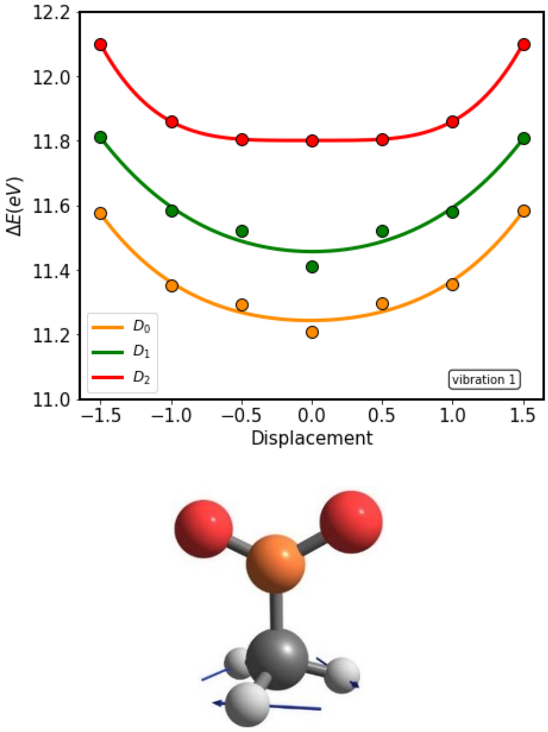

CH$_3$ twisting

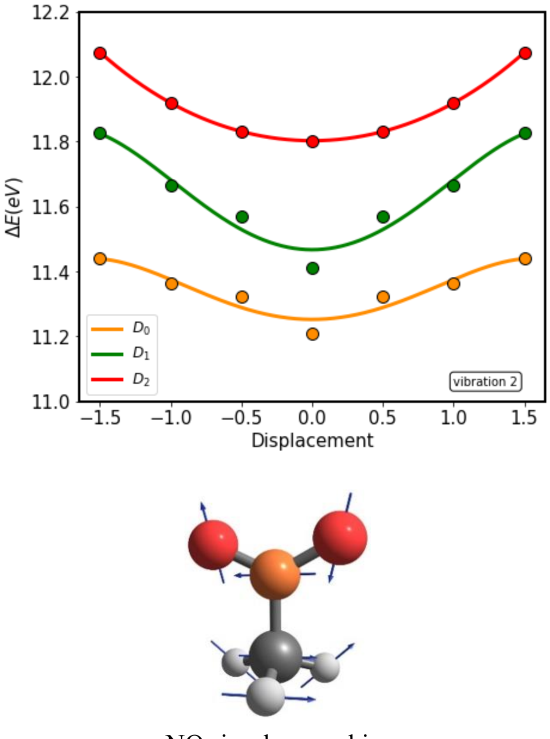

NO$_2$ in-plane rocking

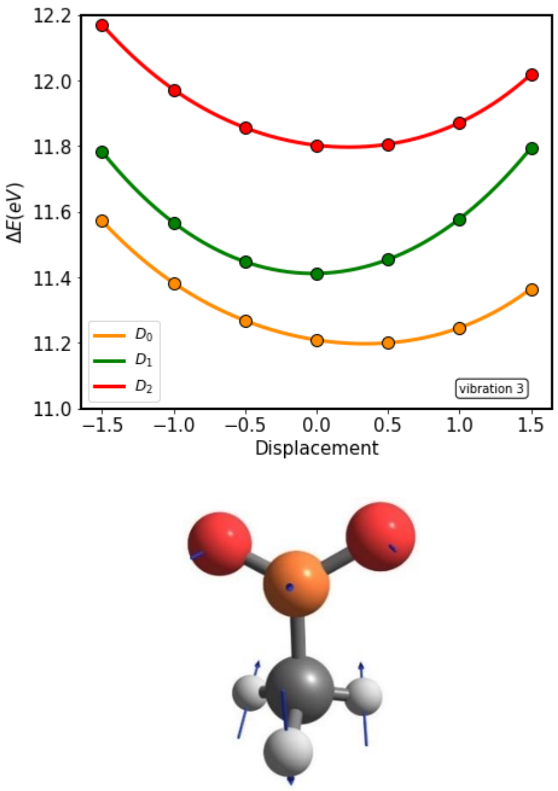

NO$_2$ out-of-plane-rocking

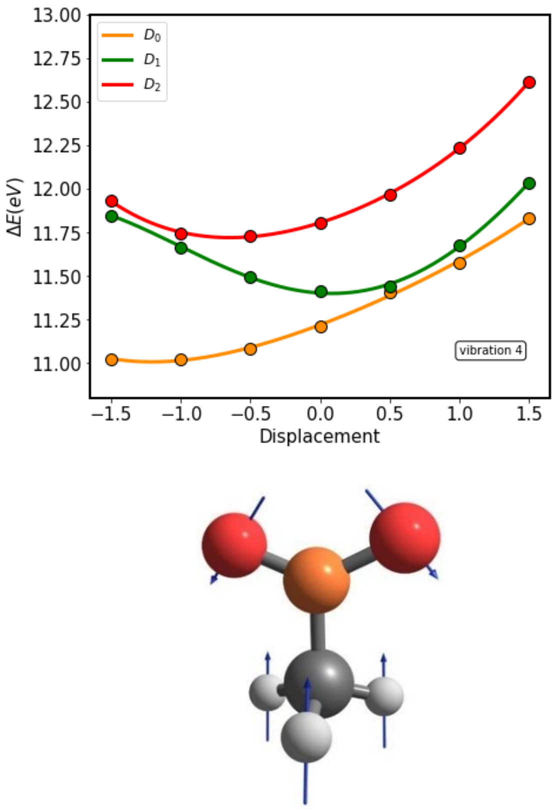

CN stretch/ sync. NO$_2$ scissoring

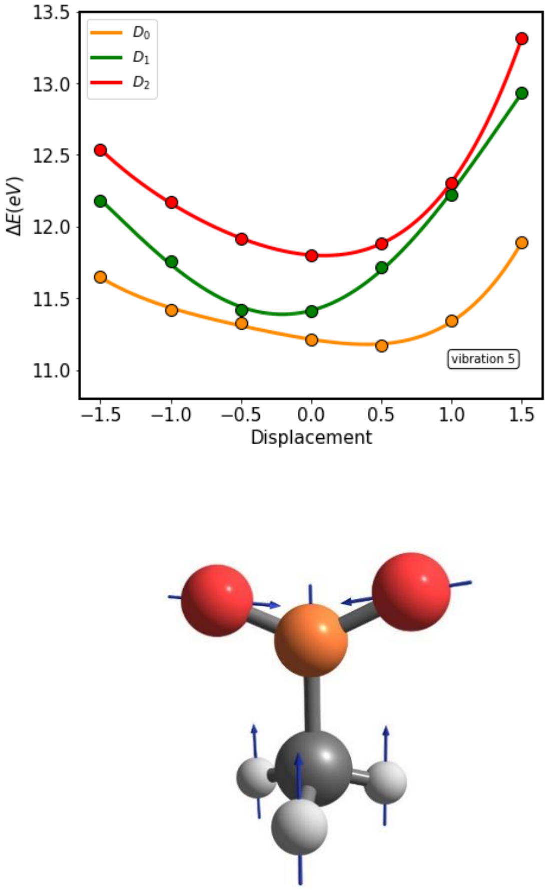

CN stretch/antisync. NO_2 scissoring

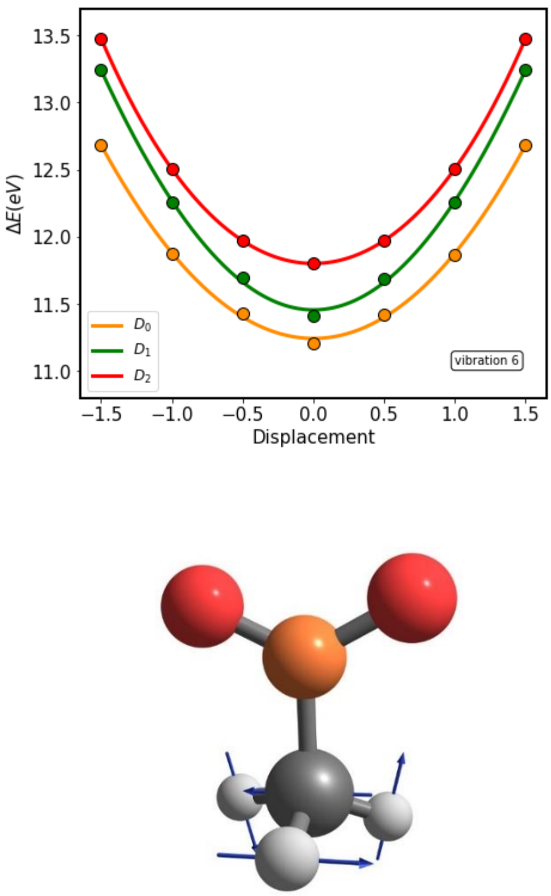

CH_3 asymmetric bending

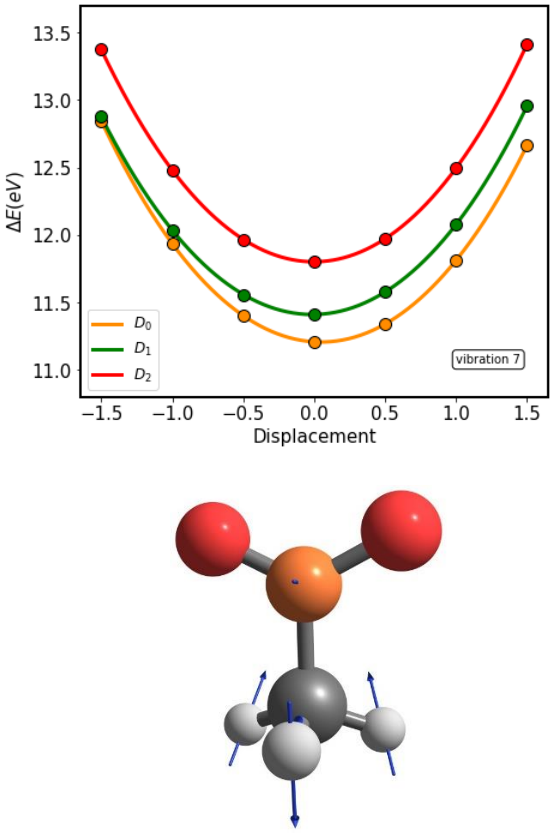

CH_3 asymmetric bending

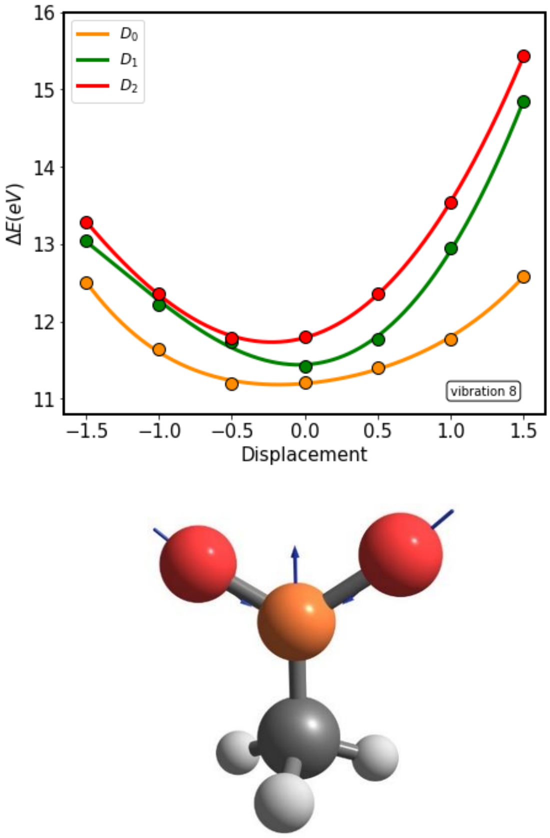

NO_2 symmetric stretching

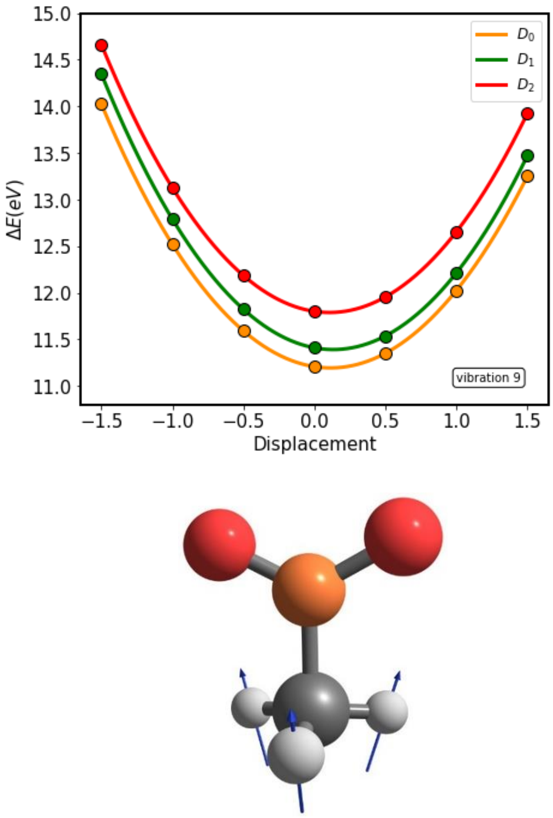

CH$_3$ symmetric bending

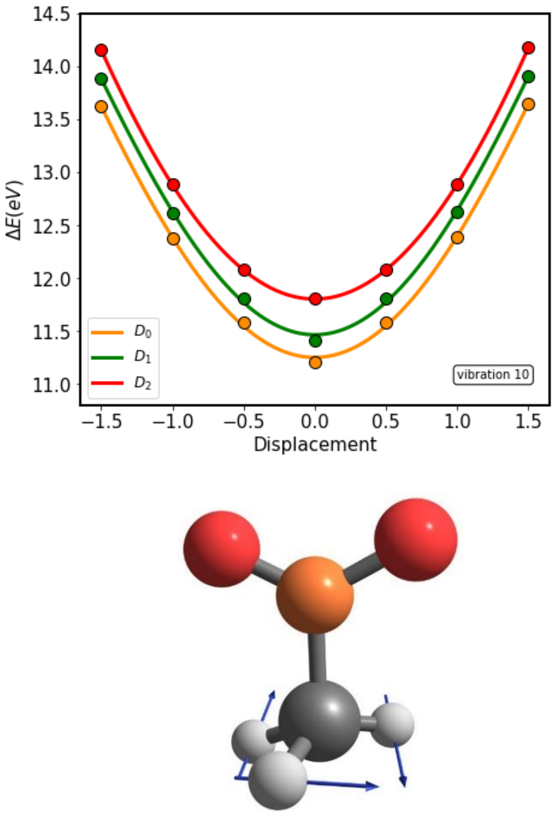

CH$_3$ asymmetric bending

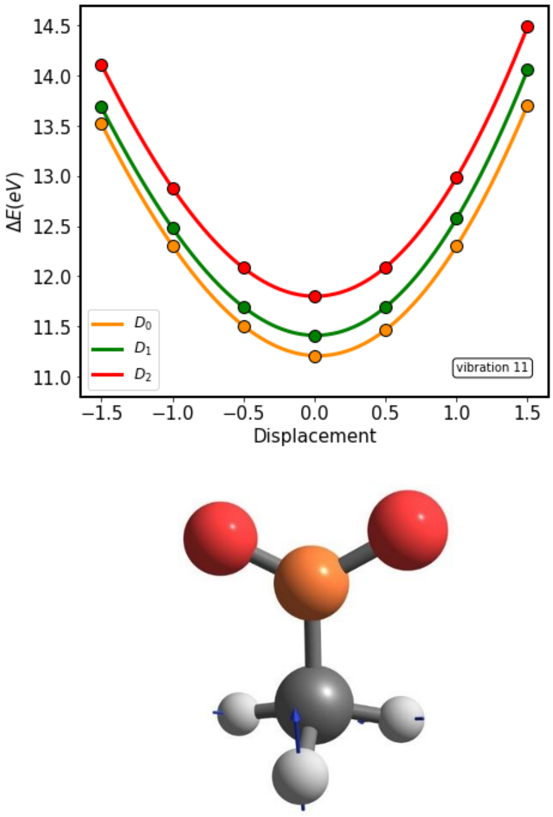

CH$_3$ symmetric bending

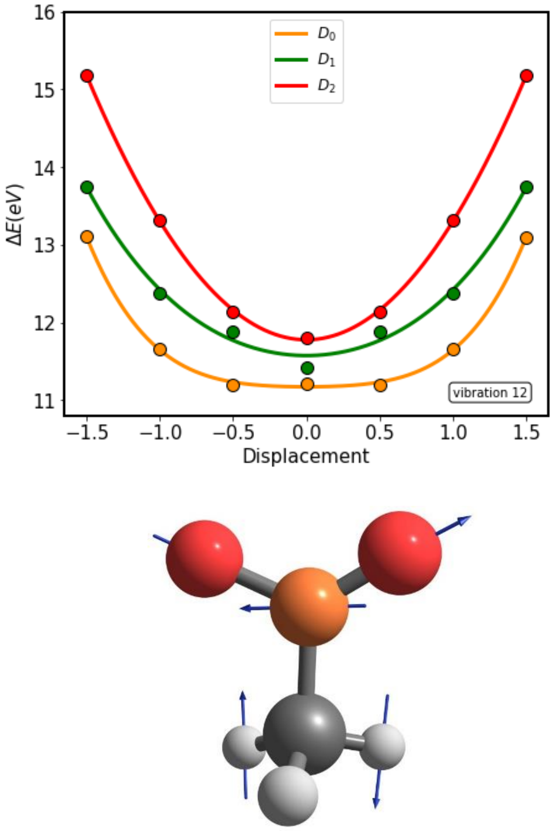

NO$_2$ asymmetric stretching

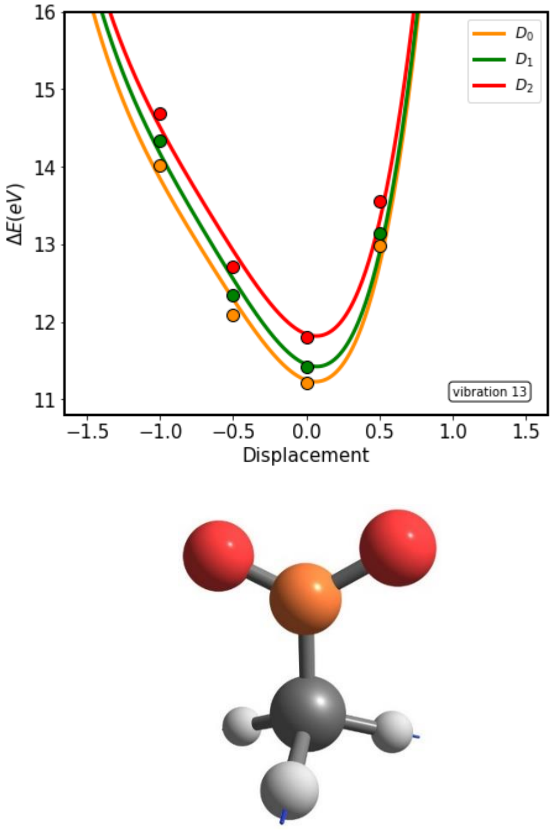

CH$_3$ symmetric stretching

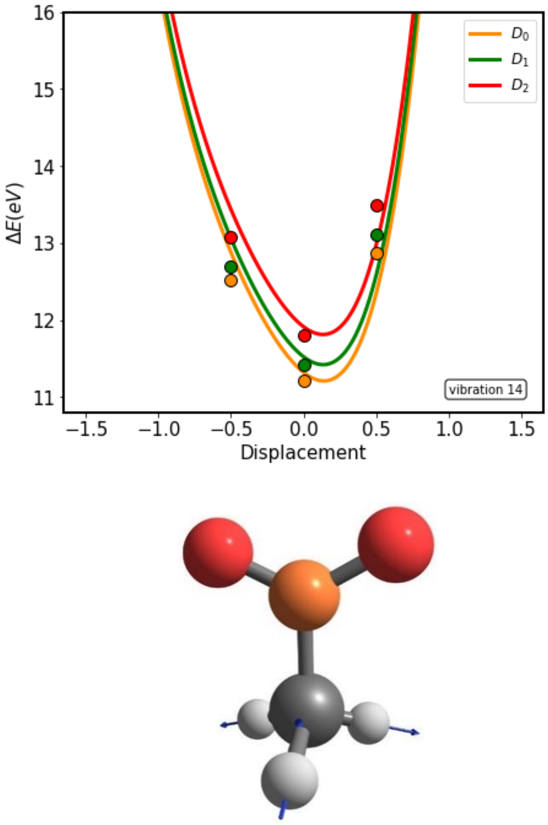

CH$_3$ asymmetric stretching

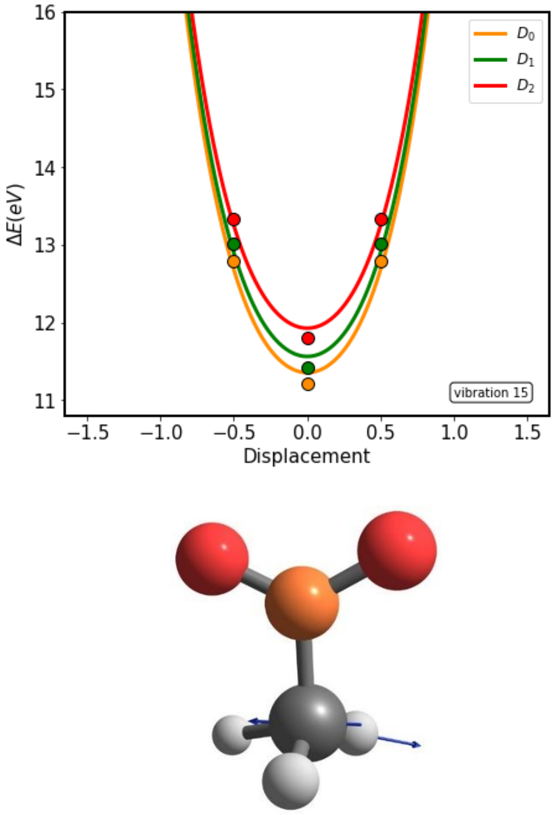

CH$_2$ symmetric stretching

Figure S4. Geometrical Parameters and Topography of the Intersection Seams

N-O distance, O-N-O angle, O-N-C-H dihedral angle, and P and B values for the different structures optimized along the C-N intersection seam for the D_0/D_1 and D_1/D_2 conical intersections.

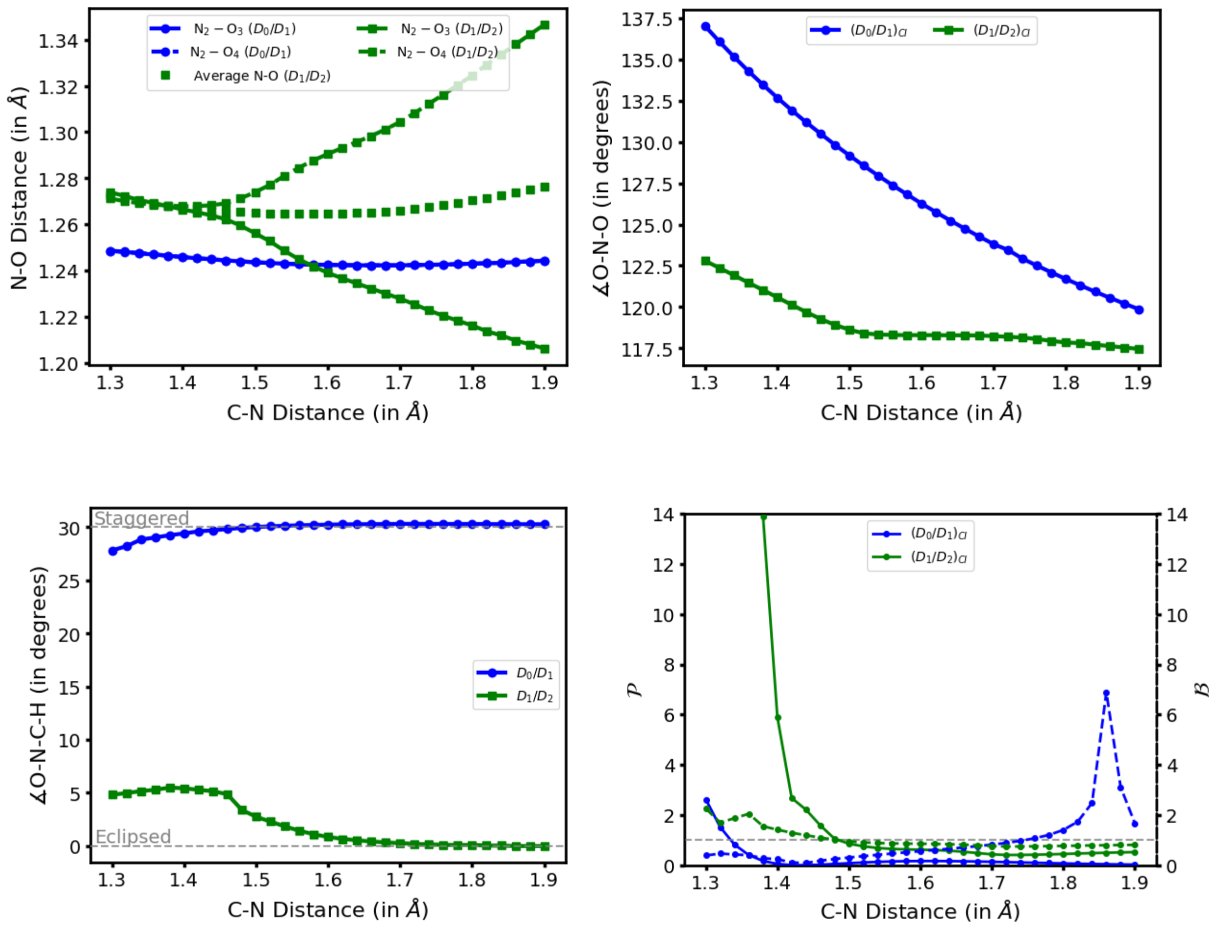

The D_1/D_2 intersection seam leads to an asymmetric stretching of the N-O bonds and favours an eclipsed conformation and a very stable (~120º) O-N-O angle. The D_0/D_1 seam, in contrast, favours the staggered conformation and is characterized by symmetric N-O bond stretching, a very large variation in the O-N-O angle (decreasing by almost ~17º) , and a large change from 1.4 to 1.9 Å in the C-N distance.

The P and B values refer to the parameters used to characterize the conical intersection topographies: P > 1 leads to a peaked intersection while P < 1 is sloped, and B > 1 is single-path while B < 1 is classed as bifurcating [I. F. Galván *et al*, J. Chem. Theory Comput. **12**, 3636 (2016)]. The right-hand side axis refers to the values of B which are represented in dashed lines (and so is the axis bar for clarity). Both seams comprise sloped and bifurcating conical intersections at C-N values similar to those corresponding to the FC region (~1.5 Å).

Figure S5. Cation Potential Energy Surfaces around the Q4 & Q5 Vibrational Coordinates

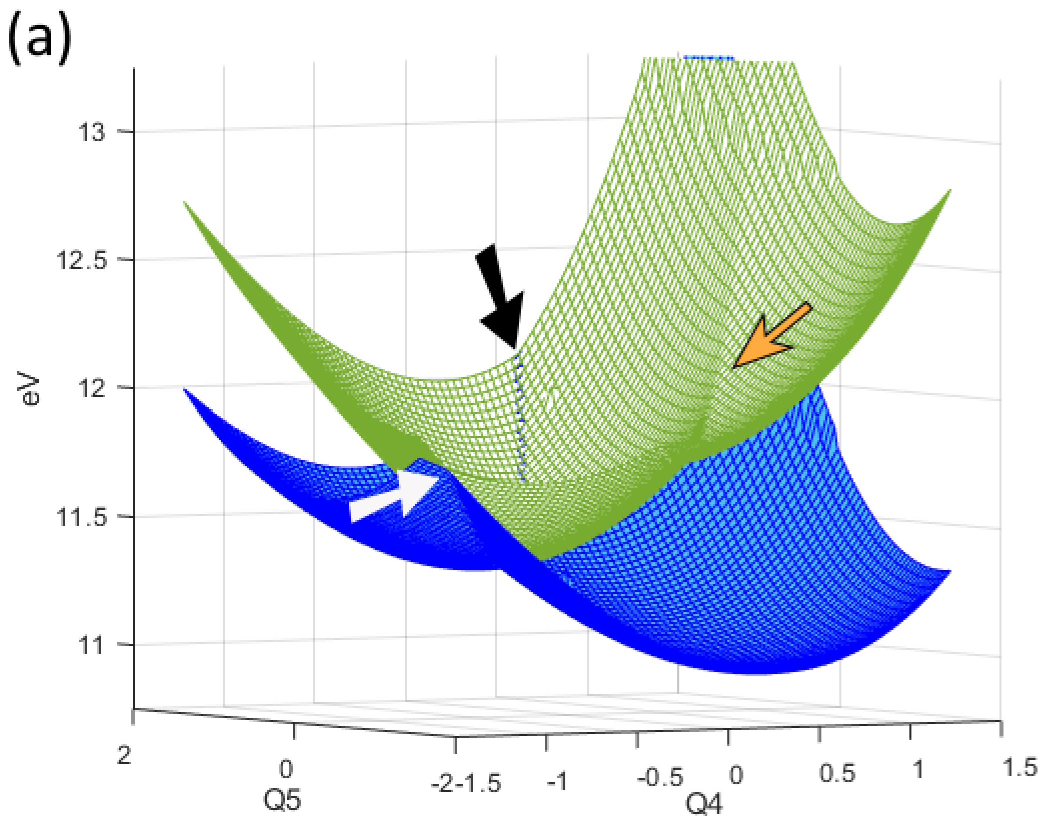

(a) (Panel above) D_0 (blue) and D_1 (green) potential energy surfaces along the Q4 and Q5 normal mode coordinates of nitromethane (equivalent motions in the cations are labelled as Q5 and Q6). Energies are relative to the S_0 ground state global minimum (in eV). The D_0/D_1 interaction seam (touching surfaces) can be seen running from the front viewing corner, as indicated by the black and white arrows. It is also possible to discern the influence of the D_1/D_2 interaction seam as a crease in the topology of the adiabatic D_1 surface (indicated by the orange arrow) caused by the avoided D_1/D_2 crossing.

(b) (Panel below). Plot showing the D_0 (blue), D_1 (green), and D_2 (orange) potential surfacess. Other details as for (a). Here the D_1/D_2 interaction seam is fully visible running between the black and white arrows and displaced from the D_0/D_1 interaction seam towards more positive Q4 distortions.

(b)

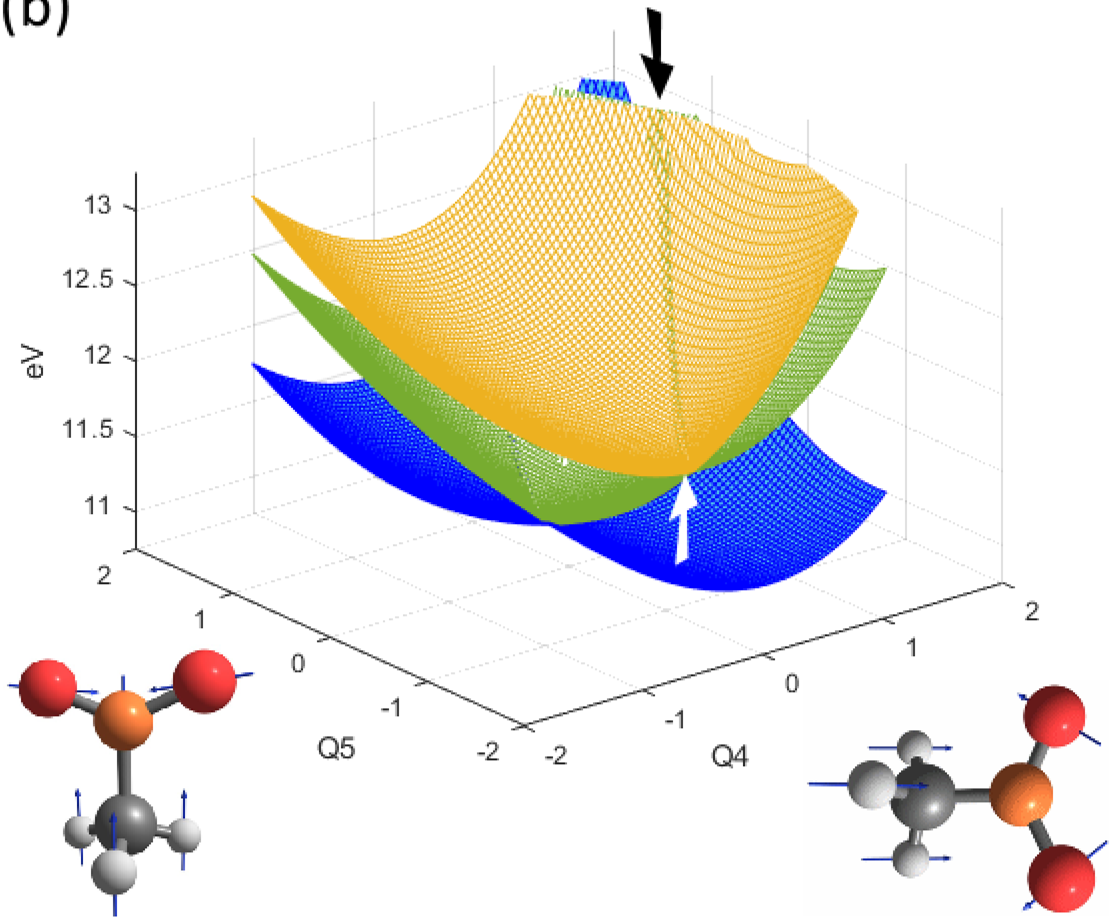

Figure S6. Energy Positioning of the Cation States around the FC Region: Torsion Angle Dependence.

An analysis of the leading CSF term in the different cation adiabatic states (i.e. D_0, D_1 and D_2) and their energy differences for C-N and O-N-O distortions at 3 representative O-N-C-H dihedral angles

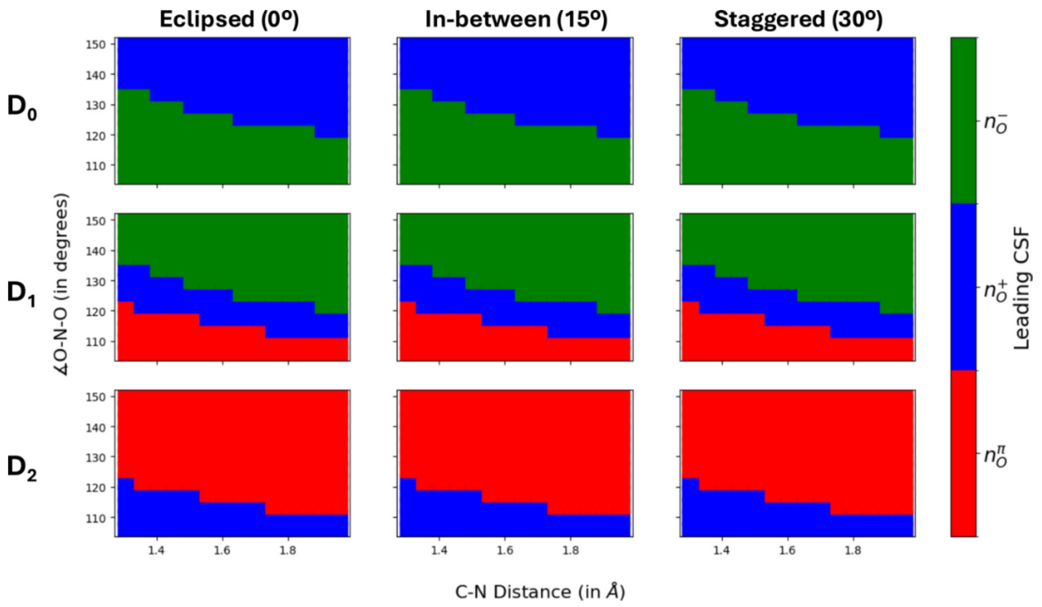

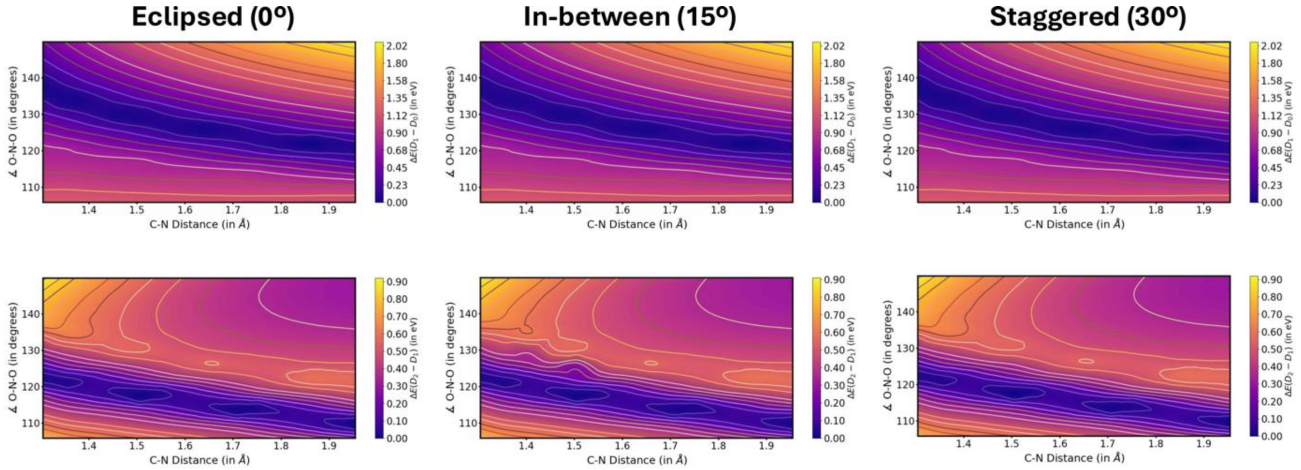

All three torsion angles computed lead to analogous D_0, D_1, D_2 cation state compositions (top 3×3 panels) and energy differences D_1-D_0, D_2-D_1 (lower 2×3 panels) which differ in the hundredth of an eV when mapped over ranges in C-N distance and O-N-O angle. The D_2/D_1 intersection seam energy along the torsion angle was also computed (inset opp.) but is found to display only negligible energy differences, validating the choice taken in the main text of focussing the discussion on the C-N and O-N-O structural parameters depicted by normal modes 4 and 5.

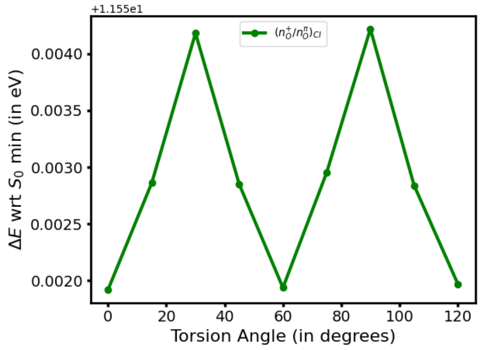

Tables

Table S1. OVGF Ionization Energies

OVGF ionization energies and pole strengths (PS) using cc-pVTZ and aug-cc-pVTZ basis sets.

	OVGF/cc-pVTZ		OVGF/aug-cc-pVTZ	
Orbital	I.E. (eV)	PS	I.E. (eV)	PS
	Staggered conformation			
8	21.201	0.86	21.331	0.858
9	18.197	0.855	18.345	0.855
10	18.043	0.817	18.007	0.864
11	17.842	0.866	18.175	0.816
12	15.898	0.919	15.987	0.918
13	15.087	0.897	15.192	0.897
14	11.601	0.891	11.834	0.889
15	11.676	0.895	11.901	0.893
16	11.561	0.887	11.798	0.884
	Eclipsed conformation			
8	21.199	0.859	21.329	0.857
9	18.171	0.855	18.319	0.855
10	18.025	0.817	17.995	0.864
11	17.831	0.866	18.156	0.816
12	15.892	0.919	15.980	0.918
13	15.083	0.897	15.189	0.896
14	11.625	0.892	11.849	0.890
15	11.644	0.894	11.881	0.892
16	11.543	0.887	11.781	0.884

Table S2. ADC(3[4+]) Ionization Energies

Comparing ADC(3[4+]) calculated ionization energies and pole strengths (PS) for staggered and eclipsed conformations, and augmented and non-augmented triple-ζ basis sets.

cc-pVTZ				aug-cc-pVTZ			
Staggered		Eclipsed		Staggered		Eclipsed	
IE (eV)	PS	IE (eV)	PS	IE (eV)	PS	IE (eV)	PS
18.375	0.726	18.373	0.724	18.504	0.714	18.501	0.712
18.026	0.737	18.025	0.735	18.162	0.717	18.161	0.714
16.911	0.503	16.908	0.502	16.984	0.490	16.981	0.489
16.070	0.907	16.070	0.907	16.141	0.905	16.141	0.905
15.519	0.006	15.522	0.006	15.645	0.006	15.648	0.006
15.372	0.000	15.374	0.000	15.510	0.000	15.511	0.000
15.101	0.009	15.098	0.009	15.226	0.009	15.222	0.009
14.950	0.002	14.952	0.003	15.090	0.001	15.090	0.002
14.854	0.699	14.854	0.699	14.955	0.704	14.954	0.704
11.963	0.886	11.963	0.886	12.123	0.883	12.123	0.883
11.771	0.882	11.773	0.882	11.929	0.878	11.930	0.878
11.498	0.868	11.497	0.868	11.672	0.864	11.671	0.864

Table S3. EOM-IP-CCSD Ionization Energies

EOM-IP-CCSD Ionization energies (eV) comparing staggered with eclipsed conformers, and augmented with non-augmented triple-ζ basis.

	cc-pVTZ		aug-cc-pVTZ	
Cation State	Staggered	Eclipsed	Staggered	Eclipsed
10	21.118	21.118	21.219	21.218
9	20.910	20.913	21.191	21.194
8	18.306	18.275	18.429	18.402
7	18.244	18.274	18.366	18.392
6	17.863	17.864	17.990	17.989
5	16.084	16.084	16.151	16.151
4	15.304	15.305	15.386	15.387
3	11.721	11.718	11.875	11.873
2	11.593	11.592	11.749	11.748
1	11.318	11.321	11.472	11.475

Table S4 CBS Extrapolations

EOM-IP-CCSD and EOM-IP-CC(2,3) calculations for vertical ionization energies (eV) at the staggered conformation using sequences of (aug-)cc-pV(X)Z (X=D, T, Q) basis sets and their extrapolation to the complete basis set (CBS) limit.

Method	n_O^-		n_O^+		n_O^π	
Basis	non-aug	aug-	non-aug	aug-	non-aug	aug-
**EOM-IP-CCSD**						
cc-pVDZ	10.86	11.23	11.16	11.54	11.29	11.70
cc-pVTZ	11.32	11.47	11.59	11.75	11.72	11.88
cc-pVQZ	11.51	11.57	11.78	11.84	11.90	11.96
CBS (∞)	11.64	11.62	11.91	11.90	12.02	12.02
**EOM-IP-CC(2,3)**						
cc-pVDZ	10.55	10.91	10.78	11.17	10.96	11.37
cc-pVTZ	10.86	11.00	11.09	11.24	11.26	11.41
cc-pVQZ [(a)]	11.00	-	11.24	-	11.40	-
CBS (∞)	11.12		11.37		11.52	

[(a)] The EOM-CC(2,3) calculation could not be converged for the aug-cc-pVQZ basis, and so these results are missing and the extrapolation to infinite basis not completed.

Table S5. Vibrational Assignments 11–12.4 eV

Energies and tentative assignments of the vibrational structure in the first two photoelectron bands of nitromethane.

CH_3NO_2 Assignment			Energy (eV)	CD_3NO_2 Assignment			Energy (eV)
D_0	P1	0 – 0	11.055	D_0	P7	0 – 0	11.059
		5^1	11.121			5^1	11.124
		5^2	11.186			5^2	11.188
		5^3	11.249		P8	6^1	11.157
	P2	6^1	11.154			$5^1 6^1$	11.218
		$5^1 6^1$	11.217	D_0	P9		11.254
	P3		11.260				11.326
			11.325				11.398
			11.388				11.472
	P4		11.297	D_0	P10		11.364
			11.360				11.429
			11.421				11.500
D_2	P5	0 – 0	11.712				11.572
		5^1	11.787	D_2	P11	0 – 0	11.716
		5^2	11.861			5^1	11.788
	P6		11.970			5^2	11.859
			12.039	D_2	P12		11.973
			12.108				12.044
			12.176				12.109
			12.240				12.177
			12.305				12.246
			12.371				

Table S6. Calculated S_0 Vibrational Frequencies

Calculated harmonic frequencies (B3LP/cc-pVTZ, with 0.97 scaling factor applied) for the CH_3NO_2 and CD_3NO_2 S_0 neutral ground states in staggered and eclipsed conformations.

Seq.	CH_3NO_2				CD_3NO_2			
	Staggered		Eclipsed		Staggered		Eclipsed	
No.	cm^{-1}	meV	cm^{-1}	meV	cm^{-1}	meV	cm^{-1}	meV
1 [(a)]	-	-	33.7	4.2	-	--	23.6	2.9
2	467.1	57.9	464.8	57.6	419.3	52.0	417.6	51.8
3	598.3	74.2	605.8	75.1	533.7	66.2	538.4	66.8
4	643.2	79.7	638.1	79.1	608.0	75.4	604.8	75.0
5	898.8	111.4	898.4	111.4	860.1	106.6	859.6	106.6
6	1076.9	133.5	1076.4	133.5	878.7	108.9	878.6	108.9
7	1102.7	136.7	1102.8	136.7	926.0	114.8	926.0	114.8
8	1358.5	168.4	1357.8	168.4	1027.9	127.4	1025.3	127.1
9	1385.9	171.8	1386.2	171.9	1030.5	127.8	1033.3	128.1
10	1421.0	176.2	1422.5	176.4	1058.5	131.2	1058.9	131.3
11	1433.7	177.8	1433.4	177.7	1378.8	170.9	1378.9	171.0
12	1585.0	196.5	1585.2	196.5	1575.7	195.4	1575.8	195.4
13	2984.0	370.0	2985.3	370.1	2136.5	264.9	2136.9	264.9
14	3068.5	380.4	3067.1	380.3	2274.8	282.0	2274.8	282.0
15	3100.2	384.4	3100.4	384.4	2305.2	285.8	2304.9	285.8

[(a)] The lowest frequency mode corresponds to the methyl torsional mode. Because of the extremely low torsional barrier this is poorly described as a harmonic vibration, and may also be reported as an unbound imaginary frequency (omitted here) when the nuclear configuration appears to sit at the top of the torsional barrier.

Table S7. Calculated D_0 Vibrational Frequencies

Calculated harmonic frequencies (B3LP/cc-pVTZ with 0.97 scaling factor applied) for the CH_3NO_2 and CD_3NO_2 D_0 $(n_O^-)^{-1}$ states in staggered and eclipsed conformations.

Seq. No.	CH_3NO_2 Staggered cm^{-1}	CH_3NO_2 Staggered meV	CH_3NO_2 Eclipsed cm^{-1}	CH_3NO_2 Eclipsed meV	CD_3NO_2 Staggered cm^{-1}	CD_3NO_2 Staggered meV	CD_3NO_2 Eclipsed cm^{-1}	CD_3NO_2 Eclipsed meV
1 [(a)]	29.7	3.7	-	-	22.2	2.8	-	-
2	252.6	31.3	254.2	31.5	239.6	29.7	241.2	29.9
3	486.8	60.4	487.1	60.4	464.4	57.6	464.9	57.6
4	529.1	65.6	531.7	65.9	473.7	58.7	475.4	58.9
5	558.2	69.2	557.7	69.1	538.8	66.8	539.0	66.8
6	834.2	103.4	834.1	103.4	813.1	100.8	810.0	100.4
7	1065.8	132.1	1065.6	132.1	849.8	105.4	854.0	105.9
8	1084.6	134.5	1085.8	134.6	904.4	112.1	903.7	112.0
9	1343.0	166.5	1338.1	165.9	978.9	121.4	976.9	121.1
10	1366.3	169.4	1379.3	171.0	990.1	122.8	993.1	123.1
11	1390.1	172.4	1382.3	171.4	1041.2	129.1	1040.6	129.0
12	1483.9	184.0	1484.0	184.0	1471.3	182.4	1471.4	182.4
13	2924.1	362.5	2926.4	362.8	2090.5	259.2	2091.2	259.3
14	3012.6	373.5	3011.2	373.3	2233.2	276.9	2233.3	276.9
15	3052.0	378.4	3051.1	378.3	2269.7	281.4	2268.8	281.3

[(a)] The lowest frequency mode corresponds to the methyl torsional mode. Because of the extremely low torsional barrier this is poorly described as a harmonic vibration, and may also be reported as an unbound imaginary frequency (omitted here) when the nuclear configuration appears to sit at the top of the torsional barrier.

Table S8. Calculated D_1 Vibrational Frequencies

Calculated D_1 $(n_O^+)^{-1}$ state harmonic frequencies (B3LP/cc-pVTZ with 0.97 scaling factor applied) for CH_3NO_2 and CD_3NO_2 in their staggered forms. [(a)]

Seq. No.	CH_3NO_2		CD_3NO_2	
	cm^{-1}	meV	cm^{-1}	meV
1 [(b)]	-	-	-	-
2	232.6	28.8	224.9	27.9
3	235.5	29.2	227.8	28.2
4	515.4	63.9	462.1	57.3
5	692.3	85.8	537.2	66.6
6	745.0	92.4	665.5	82.5
7	829.2	102.8	690.0	85.5
8	1217.8	151.0	947.5	117.5
9	1280.1	158.7	1006.4	124.8
10	1368.3	169.7	1009.2	125.1
11	1375.9	170.6	1239.5	153.7
12	1635.0	202.7	1632.4	202.4
13	2975.3	368.9	2109.4	261.5
14	3140.6	389.4	2341.6	290.3
15	3155.7	391.3	2354.2	291.9

(a) D_1 geometry optimisations for the eclipsed form did not converge. Hence normal mode frequencies could not be obtained.

(b) The lowest frequency mode corresponds to the methyl torsional mode. Because of the extremely low torsional barrier this is poorly described as a harmonic vibration, and may also be reported as an unbound imaginary frequency (omitted here) when the nuclear configuration appears to sit at the top of the torsional barrier.

Table S9. Calculated D_2 Vibrational Frequencies

Calculated harmonic frequencies (B3LP/cc-pVTZ with 0.97 scaling factor applied) for the CH_3NO_2 and CD_3NO_2 D_2 $(n_O{}^{\pi})^{-1}$ states in staggered and eclipsed conformations.

Seq. No.	CH_3NO_2 Staggered cm^{-1}	CH_3NO_2 Staggered meV	CH_3NO_2 Eclipsed cm^{-1}	CH_3NO_2 Eclipsed meV	CD_3NO_2 Staggered cm^{-1}	CD_3NO_2 Staggered meV	CD_3NO_2 Eclipsed cm^{-1}	CD_3NO_2 Eclipsed meV
1 [(a)]	-	-	128.0	15.9	-	-	94.1	11.7
2	219.0	27.1	414.7	51.4	189.4	23.5	372.0	46.1
3	441.7	54.8	486.6	60.3	404.1	50.1	472.1	58.5
4	538.9	66.8	523.8	64.9	484.4	60.1	483.3	59.9
5	593.8	73.6	617.1	76.5	571.1	70.8	590.4	73.2
6	832.4	103.2	856.9	106.2	821.3	101.8	826.4	102.5
7	1090.5	135.2	1069.1	132.6	896.6	111.2	884.8	109.7
8	1119.3	138.8	1134.6	140.7	905.4	112.3	941.2	116.7
9	1301.0	161.3	1326.2	164.4	988.8	122.6	974.1	120.8
10	1381.9	171.3	1380.0	171.1	990.8	122.8	993.2	123.1
11	1389.3	172.3	1402.4	173.9	1048.0	129.9	1048.1	129.9
12	1410.2	174.8	1436.6	178.1	1335.1	165.5	1418.3	175.9
13	2930.7	363.4	2930.6	363.3	2101.3	260.5	2096.9	260.0
14	3033.1	376.1	3001.1	372.1	2241.4	277.9	2223.7	275.7
15	3093.9	383.6	3096.3	383.9	2304.0	285.7	2303.7	285.6

[(a)] The lowest frequency mode corresponds to the methyl torsional mode. Because of the extremely low torsional barrier this is poorly described as a harmonic vibration, and may also be reported as an unbound imaginary frequency (omitted here) when the nuclear configuration appears to sit at the top of the torsional barrier.
